# Gut Microbiota Has a Crucial Role in the Development of Hypertension and Vascular Dysfunction in Toll-like Receptor 7-Driven Lupus Autoimmunity

**DOI:** 10.3390/antiox10091426

**Published:** 2021-09-07

**Authors:** Néstor de la Visitación, Iñaki Robles-Vera, Javier Moleón, Cristina González-Correa, Nazaret Aguilera-Sánchez, Marta Toral, Manuel Gómez-Guzmán, Manuel Sánchez, Rosario Jiménez, Natividad Martin-Morales, Francisco O’Valle, Miguel Romero, Juan Duarte

**Affiliations:** 1 Department of Pharmacology, School of Pharmacy and Center for Biomedical Research (CIBM), University of Granada, 18071 Granada, Spain; nestorvp@correo.ugr.es (N.d.l.V.); irv1991@correo.ugr.es (I.R.-V.); javiermm95@ugr.es (J.M.); cristinaglez@ugr.es (C.G.-C.); nazaretaguilera@correo.ugr.es (N.A.-S.); mgguzman@ugr.es (M.G.-G.); manuelsanchezsantos@ugr.es (M.S.); rjmoleon@ugr.es (R.J.); miguelr@ugr.es (M.R.); 2 Gene Regulation in Cardiovascular Remodeling and Inflammation Group, Centro Nacional de Investigaciones Cardiovasculares (CNIC), 28029 Madrid, Spain; 3 Ciber de Enfermedades Cardiovasculares (CIBERCV), 28029 Madrid, Spain; 4 Instituto de Investigación Biosanitaria de Granada, ibs.GRANADA, 18007 Granada, Spain; fovalle@ugr.es; 5 Department of Pathology, School of Medicine, University of Granada, 18007 Granada, Spain; nati@ugr.es

**Keywords:** hypertension, endothelial dysfunction, gut dysbiosis, immune system, systemic lupus erythematosus, TLR7 activation

## Abstract

Our group has investigated the involvement of gut microbiota in hypertension in a murine model of systemic lupus erythematosus induced by Toll-like receptor (TLR)-7 activation. Female BALB/c mice were randomly assigned to four experimental groups: an untreated control (CTR), a group treated with the TLR7 agonist imiquimod (IMQ), IMQ-treated with vancomycin, and IMQ-treated with a cocktail of broad-spectrum antibiotics. We carried out faecal microbiota transplant (FMT) from donor CTR or IMQ mice to recipient IMQ or CTR animals, respectively. Vancomycin inhibited the increase in blood pressure; improved kidney injury, endothelial function, and oxidative stress; and reduced T helper (Th)17 infiltration in aortas from IMQ-treated mice. The rise in blood pressure and vascular complications present in IMQ mice were also observed in the CTR mice recipients of IMQ microbiota. Reduced relative populations of *Sutterella* and *Anaerovibrio* were associated with high blood pressure in our animals, which were increased after stool transplantation of healthy microbiota to IMQ mice. The reduced endothelium-dependent vasodilator responses to acetylcholine induced by IMQ microbiota were normalized after interleukin-17 neutralization. In conclusion, gut microbiota plays a role in the TLR7-driven increase in Th17 cell, endothelial dysfunction, vascular inflammation, and hypertension. The vascular changes induced by IMQ microbiota were initiated by Th17 infiltrating the vasculature.

## 1. Introduction

Systemic lupus erythematosus (SLE) is among the most deleterious autoimmune inflammatory diseases, in which the synthesis of autoantibodies is promoted, forming immune complexes, that in turn deposit in target organs, causing damage to the tissues. SLE is related to an increased risk of renal and cardiovascular disease development [1], the most important cause of mortality in SLE patients [2]. It mainly affects young women of child-bearing age. This disease is linked to an elevated incidence of hypertension [3]. Environmental, genetic, metabolic, and hormonal element interaction partakes in SLE predisposition [4]. However, the pathophysiological mechanisms responsible for the onset of SLE hypertension are not comprehended yet in their entirety. Inflammatory cytokines and reactive oxygen species (ROS) contribute to the pathogenesis of SLE hypertension. These mediators are known local pro-inflammatory stimuli that trigger the vascular and renal dysfunction, possibly occurring downstream of the early immune system dysregulation [5].

Toll-like receptors (TLRs) are a family of innate pattern recognition receptors that recognize a broad spectrum of pathogen-associated molecular patterns, triggering innate immune response mechanisms [6]. There is substantial proof of their pathological role in the onset and progression of human and spontaneous mouse models of SLE [7,8]. TLR7 activation can lead to phenotypic and functional shifts seen in human SLE, such as high autoantibody levels and multiple organ involvement [9]. In fact, increased TLR7 gene copies, single nucleotide polymorphisms, and up-regulation of signalling pathways downstream of TLR7 have been associated with human SLE susceptibility [10]. The TLR7/interferon (IFN) signalling pathway is key in the genesis of human SLE [11]. In female NZBWF1 mice, which have multiple lupus susceptibility loci and weak IFN signature, dual TLR7 and TLR9 antagonism partially prevented the disease progression [12]. Recently, we demonstrated that TLR7 activation causes hypertension and vascular damage in BALB/c mice, and emphasizes the elevated vascular inflammation and oxidative stress, mediated in part by interleukin (IL)-17, as crucial factors for cardiovascular complications [13].

New studies are showing that gut microbiota composition is linked to the pathogenesis of SLE. Intestinal microbiota may trigger symptoms and exacerbate this pathology in both murine models of SLE and patients [14,15,16,17,18,19,20,21,22,23,24]. We recently demonstrated in female NZBWF1 (F1 hybrid of New Zealand Black and New Zealand White strains) mice that gut microbiota changes are linked to hypertension [25]. Interestingly, gut dysbiosis was found in TLR7-dependent mouse models of SLE, and bacterial translocation of *Lactobacillus reuteri* to secondary lymphoid tissue and liver can drive autoimmunity, which was improved with dietary resistant starch by suppressing the pathological levels of L. reuteri and its translocation, through short-chain fatty acids (SCFAs) [22]. We recently demonstrated that probiotics consumption reduced BP under TLR7 activation conditions [26]. In spite of all the data associating intestinal dysbiosis and autoimmunity in TLR7-dependent lupus mice (gut microbiota depletion ameliorates the IFN pathway and autoimmunity), no information is available on the involvement of the microbiota in the pathogenesis of hypertension and vascular alterations in these animals. Previous studies on germ-free mouse models demonstrated the contributory role of gut microbiota in vascular inflammation, vascular dysfunction, and blood pressure (BP) regulation [27,28,29]. In this study, our group investigated the hypothesis that gut microbiota is able to enhance the predisposition to SLE onset and T cell maturation in intestinal secondary lymphoid tissues, triggering a loss of endothelial function and hypertension in a lupus model induced by epicutaneous application of the TLR7 agonist imiquimod (IMQ).

## 2. Materials and Methods

### 2.1. Animals and Experimental Groups

All animal procedures performed conformed to the guidelines from Directive 2010/63/EU of the European Parliament on the protection of animals used for scientific purposes and the current NIH guidelines, and received the approval from the Ethics Committee of Laboratory Animals of the University of Granada (Spain) (Ref. 12/11/2017/164). Additionally, we followed the Guidelines for Transparency on Gut Microbiome Studies in Essential and Experimental Hypertension [30], and are compliant with the ARRIVE guidelines [31,32]. Seven- to nine-weeks-old female BALB/c mice, were purchased from Janvier (Le Genest, France). To assess the role of gut microbiota, we reduced bacterial mass using antibiotic treatments. Mice were randomly sorted into four experimental groups of 6–8 animals each: an untreated control (CTR) (*n* = 7), a group treated with IMQ (IMQ) (*n* = 6), IMQ-treated with vancomycin (2 g·L^−1^) (IMQ-VANCO) (*n* = 8), and IMQ-treated with a broad-spectrum cocktail of antibiotics (IMQ-MIX) (*n* = 7). Vancomycin is a molecule that cannot be absorbed in the intestines and eliminates mainly Gram-positive bacteria. In order to examine the role of vancomycin-resistant bacteria in BP control we prepared a cocktail of un-absorbable antibiotics with a broad-spectrum, including vancomycin. The cocktail of antibiotics used was a mixture of vancomycin (0.5 g·L^−1^; Pfizer), metronidazole (1 g·L^−1^; Sigma), neomycin (1 g·L^−1^; Fisher Scientific), and ampicillin (1 g·L^−1^; Sigma) in drinking water [21]; in addition to the antibiotics, a sweetener (Equal, 4 g·L^−1^) was used to overcome the metallic taste of metronidazole, for control purposes, it was also added to the rest of experimental groups. A total of 1.25 mg of 5% IMQ cream (Aldara^®^) from Laboratories MEDA PHARMA SALU (Madrid, Spain) was applied topically on the right ears of IMQ-treated mice three times in alternate days per week for 8 weeks. IMQ has been used to treat various skin neoplasms, including genital warts, actinic keratoses, and superficial basal carcinomas. However, there has been no case described in the literature in which the development of SLE resulted from IMQ treatment. This fact seems to be related to the dose of IMQ used for mice, which was 200-fold higher than the clinical dose used in humans. Moreover, compared with intraperitoneal administration of IMQ, topical application of this drug to the skin efficiently promotes systemic autoimmune disease [9], which justifies the topical use of IMQ in our experiments.

Due to their increased predisposition to TLR7-driven functional responses and autoimmunity [33], we used female mice. In order to analyse the effects of antibiotics in control mice we tested the effects VANCO (CTR-VANCO) or MIX (CTR-MIX) in normotensive mice, without topical IMQ application (*n* = 8).

In order to investigate if the microbiota in the IMQ model is linked to BP regulation, an FMT was carried out (Figure S1), following previously used protocols [34]. For this reason, stool samples were freshly obtained from individual IMQ and CTR mice at 8 weeks. The samples were pooled and suspended in a 1:20 (*w*/*v*) solution in sterile phosphate-buffered saline (PBS) and superfluous faecal material was sedimented and discarded at 60 g for 5 min. The suspension was collected in aliquots and kept at −80 °C until used. IMQ-treated for 8 weeks and aged-matched CTR female mice were used as recipient mice. These mice were administered with ceftriaxone sodium (400 mg Kg^−1^) once daily for 5 consecutive days by oral gavage. The purpose of this antibiotic treatment was to reduce the pre-existing bacteria populations to boost the proliferation in the host of intestinal microorganisms from donor animals post-FMT. After two days from the end of the treatment with ceftriaxone, recipient mice received already prepared faecal suspensions (0.1 mL) for 3 consecutive days through oral gavage, and later once every 3 days for a total period of 2 weeks. Animals were randomly assigned to four different groups of 8 animals each: control with control microbiota (CTR-CTR), control with IMQ microbiota (CTR-IMQ), IMQ with IMQ microbiota (IMQ-IMQ), and IMQ with control microbiota (IMQ-CTR). IMQ-recipient mice were also treated with IMQ cream, as was described above.

For our procedures, the animals were maintained in specific pathogen-free (SPF) installations at the University of Granada Biological Services Unit under standard conditions (12 h light and dark cycles, 21–22 °C, 60 ± 10% humidity). Makrolom cages (Ehret, Emmerdingen, Germany) with dust-free laboratory bedding and enrichment were used to house the mice in separate cages to avoid horizontal transmission of the microbiota among them. The animals received water and standard laboratory diet (SAFE A04, Augy, France) ad libitum. Water bottles were renewed daily, and both water and food intakes were studied and controlled on a day-to-day basis for all groups. Animals were stochastically sorted into groups and the experimenter was blinded to drug treatment until data analysis had been performed.

### 2.2. Blood Pressure, Morphology, Organ Weight Indices, and Renal Injury

SBP was obtained from conscious mice through tail-cuff plethysmography (Digital Pressure Meter, LE 5001; Letica S.A., Barcelona, Spain). A minimum of seven replicates (per mouse) were recorded per session and the mean of the lowest three values within 5 mmHg was considered the SBP level [35].

Body weight (in grams) was determined for all mice. After every experimental endpoint, mice were euthanized under inhalation anaesthesia with 2% isoflurane. The hearts were excised; the atria and the right ventricle were then discarded to obtain the weight of the left ventricle. Organ weight indices were considered through their ratio with the tibia length for normalization. All samples were snap-frozen in liquid nitrogen and kept at −80 °C until used.

Our protocols for the study of kidney histology are extensively described in previous documents [35]. After fixation and paraffination, transversal sections were obtained and stained with Mason’s Trichrome and Haematoxylin–Eosin. Evaluation of lesions at kidney, glomeruli, and tubulointerstitial spaces levels was performed after a semiquantitative fashion.

### 2.3. Plasma and Urine Parameters

Blood samples were extracted from the left ventricle, cooled on ice, and centrifuged for 10 min at 3500 rpm at 4 °C. Next, plasma samples were kept at −80 °C. Plasma anti-ds-DNA and anti-cardiolipin antibody levels were determined with a mouse Anti-dsDNA IgG ELISA Kit (Alpha Diagnostic International, San Antonio, TX, USA), and a mouse Anti-cardiolipin total Ig (IgG + IgA + IgM) ELISA Kit (Alpha Diagnostic International, San Antonio, TX, USA), respectively, following the manufacturer’s instructions, as previously described [36]. Plasma cytokines were measured with a multiplex assay through Luminex technology (Merck Millipore, Darmstadt, Germany). Combur Test strips (Roche Diagnostics, Mannheim, Germany) were used to quantify proteinuria.

### 2.4. Vascular Reactivity Studies

Descending thoracic aortic samples were prepared from animals and placed in a wire myograph (model 610 M, Danish Myo Technology, Aarhus, Denmark) with Krebs solution (composition in mM: 118 NaCl, 4.75 KCl, 25 NaHCO_3_, 1.2 MgSO_4_, 2 CaCl_2_, 1.2 KH_2_PO_4_ and 11 glucose) at 37 °C and gassed with 95% O_2_ and 5% CO_2_ (pH = 7.4) for isometric tension quantification as described in previous articles [37]. Length-tension parameters were assessed with Myodaq 2.01 software and the tissue segments were pre-tensed to a tension of 5 mN.

In aortic whole tissue rings, cumulative concentration–response functions to acetylcholine (ACh, 1 Nm–10 μM) were performed precontracting with the thromboxane A_2_ analogous U46619 (10 nM). Posterior curves to ACh were carried out in each aortic segment with the specific pan-NOX inhibitor VAS2870 (1 µM), or the Rho kinase inhibitor Y27632 (1 µM) plus their respective controls. In order to test the role of IL-17, we coincubated aorta segments for 6 h with anti-IL-17a antibody (10 µg·L^−1^) before performing the concentration-response curves to ACh. All curves are shown as a percentage in relation to precontraction tension with U46619.

### 2.5. NADPH Oxidase Activity and eNOS Expression

We employed the lucigenin-enhanced chemiluminescence assay to evaluate NADPH oxidase activity in samples of aorta as previously described [38]. Segments from all animals were incubated for 30 min at 37 °C in a physiological saline solution (pH 7.4) of the following composition (in mM): NaCl 119, HEPES 20, KCl 4.6, MgSO_4_ 1, Na_2_HPO_4_ 0.15, KH_2_PO_4_ 0.4, NaHCO_3_ 1, CaCl_2_ 1.2 and glucose 5.5. Aortic production of O_2_^−^ was triggered with NADPH (100 μM), additional tubes without NADPH were used to measure the baseline response. Lucigenin was injected automatically at a final concentration of 5 μM to reduce the chance of artifacts that normally occur at higher concentration levels. NADPH oxidase activity was ascertained through luminescence measurements over 200 s in a scintillation counter (Lumat LB 9507, Berthold, Germany) in 5-s intervals and represented by subtracting the baseline signal from the one obtained with NADPH. Dry tissue weight was recorded for all samples. NADPH oxidase activity is expressed as relative luminescence units (RLU) min^−1^ mg^−1^ dry aortic tissue.

### 2.6. Flow Cytometry

We obtained MLNs, spleens, blood, and aorta from all animals. The tissues were homogenized with slides and then wetted to decrease friction. Cells were isolated filtering them through 40-µm cell strainers. Then, the red blood cells were lysed with Gey’s solution. For a better assessment of intracellular markers, 1 × 10^6^ cells per sample and panel were incubated with a protein transport inhibitor (BD GolgiPlug^TM^), concomitantly, cell cytokine synthesis was stimulated with 50 ng·L^−1^ phorbol 12-myristate 13-acetate plus 1 μg·L^−1^ ionomycin. After 4.5 h, the cells were blocked with anti-Fc-γ receptor antibodies to avoid nonspecific links to these receptors (Miltenyi Biotec) and were stained with a live/dead kit as a viability assay (LIVE/DEAD^®^ Fixable Aqua Dead Cell Stain, Thermo Fisher), incubating for 30 min at 4 °C in PBS. After, cells were stained for surface markers for 20 min at 4 °C in the dark with anti-CD3 (PE, clone REA641 Miltenyi) anti-CD4 (PerCP-Cy5.5, clone RM4-5 Invitrogen), anti-CD25 (PE-VIO770, clone 7D4 Miltenyi), anti-CD45 (FITC, clone 30-F11 Miltenyi), and anti-B220 (APC, clone RA3-6B2, BD Bioscience) in flow cytometry staining buffer (FCS, PBS, 1% bovine serum albumin), depending on the panel studied. Cells were then fixed and subsequently permeabilized through consecutive incubations separated by a wash with PBS using the buffers A and B, respectively (Fisher Scientific, Madrid, Spain), and intracellular staining was conducted for 30 min at 4 °C in the dark with anti-IFN-γ (PE-VIO770, clone XMG1.2, eBioscience, San Diego, CA, USA) and anti-IL-17a (PE-Cy7, clone eBio17B7, eBioscience, San Diego, CA, USA) for their respective panels. Finally, cells were resuspended in test tubes with FCS buffer. Sample analysis was carried out with a flow cytometer Canto II (BD Biosciences) as previously described [37,38]. The gating strategy used is shown in Figure S2.

### 2.7. DNA Extraction, 16S rRNA Gene Amplification, Bioinformatics

As described in previous articles [25], stools from all groups (*n* = 6–8) were stored at −80 °C. Microbial DNA was obtained with G-spin columns (INTRON Biotechnology) from 30 mg of faecal content in suspension with PBS, digested using proteinase K and RNAses, assessing resulting DNA concentrations with Quant-IT PicoGreen (Thermo Fischer). Aliquots from our samples (approximately 3 ng) were utilized to amplify the V3-V4 region of the 16S rRNA gene [39]. PCR products (approximately 450 bp) included extension tails, which allowed sample barcoding and the addition of specific Illumina sequences in a second low-cycle number PCR. Individual amplicon libraries were analysed with a Bioanalyzer 2100 (Agilent) and a pool of samples was made in equimolar amounts. The pool was further cleaned, quantified and the exact concentration estimated through real time PCR (Kapa Biosystems). Finally, we sequenced the samples with an Illumina MiSeq instrument with 2 × 300 paired-end read sequencing at the Unidad de Genómica (Parque Científico de Madrid, Madrid, Spain).

Raw sequences were processed with the barcoded Illumina paired-end sequencing (BIPES) pipeline [40]. First, the barcode primers were discarded when containing ambiguous bases or mismatches in the primer regions following BIPES protocols. Second, we eliminated all sequences found with more than one mismatch within the 40–70 bp region at each end. Third, we utilized 30 Ns to concentrate the two single-ended sequences for the downstream sequence analysis. The description in detail of the process has been previously published [41]. Fourth, we utilized UCHIME (implemented in USEARCH, version 6.1) in order to remove chimeras in the de novo mode (using-minchunk 20-xn 7-noskipgaps 2) [42].

Between 90,000 and 220,000 sequences were identified in each sample. All ensuing analyses were carried out with 16S Metagenomics (Version: 1.0.1.0) from Illumina. The sequences were then clustered to an operational taxonomic unit (OTU) with USEARCH employing default parameters (USERACH61). The threshold distance was set to 0.03. Hence, when the similarity between two 16S rRNA sequences was 97%, the sequences were classified as the same OTU. QIIME-based alignments of representative sequences were carried out with PyNAST, and the Greengenes 13_8 database was utilized as the template file. The Ribosome Database project (RDP) algorithm was applied to classify the representative sequences into specific taxa with the default database [43]. The Taxonomy Database (National Center for Biotechnology Information) was utilized for classification and nomenclature. Bacteria were classified based on the SCFAs end-product as previously described [44]. 

### 2.8. Statistical Analysis

The microecological parameters described in this experiment were determined with Palaeontological Statistics (PAST 4×). Reads in each OTU were normalized to total reads in each sample. Only taxa with a percentage of reads >0.001% were utilized in the analysis. Principal components analysis (PCA) analyses were also performed with these data to identify significant differences between groups, using PAST 4.02, and SSPS. LDA scores above 3.5 were displayed, and Kruskal–Wallis test among classes and Wilcoxon test between subclasses with threshold 0.05. Taxonomy was uploaded to the Galaxy platform [45] to generate Linear discriminant analysis effect size (LEfSe)/cladogram enrichment plots considering significant enrichment at a *p* < 0.05. A volcano plot was generated by plotting −log10 of the *p*-value against the log2 fold change of genus with the R package, ggplot2 [46]. A quasi-likelihood F-test transformed *p*-value threshold (*p* < 0.05) was utilized to highlight significantly differentiating data points. All data were processed with GraphPad Prism 8. Results are represented as means ± SEM. The progression of tail SBP and the ex vivo vascular reactivity assays analysis was performed through two-way analysis of variance (ANOVA) with the Tukey post hoc test. The rest of the variables were tested on normal distribution using Shapiro–Wilk normality test and compared with an unpaired *t* test or one-way ANOVA and Tukey post hoc test in case of normal distribution, or Mann–Whitney test or Kruskal–Wallis with Dunn’s multiple comparison test in case of abnormal distribution. *p* < 0.05 was considered statistically significant.

## 3. Results

### 3.1. Antibiotic Treatment Prevented the Raise of Blood Pressure, Renal Injury, and Disease Activity in TLR7-Dependent SLE

**Figure 1:**
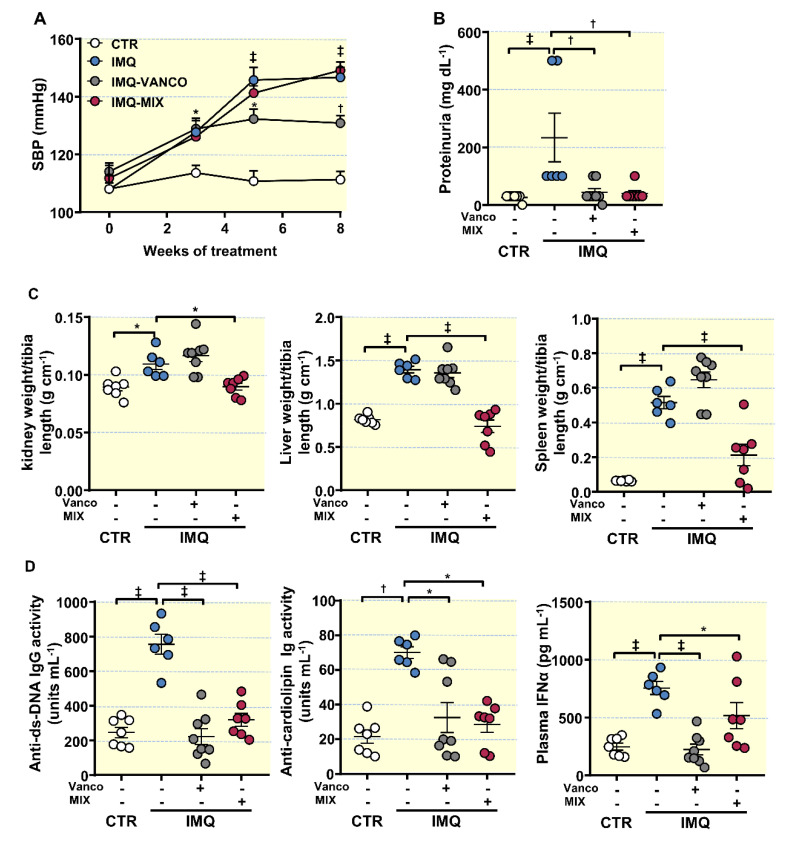
Effects of microbiota modification through population depletion with antibiotics on blood pressure, proteinuria, morphology, and disease activity in the imiquimod (IMQ) group. (**A**) Systolic blood pressure (SBP) assessed with tail-cuff plethysmography, (**B**) proteinuria, (**C**) organ hypertrophy levels, (**D**) circulating double-stranded DNA (anti-ds-DNA) and anti-cardiolipin autoantibodies, and interferon (IFN)α levels in control (CTR), IMQ and IMQ-groups treated with Vancomycin (VANCO) or a cocktail of antibiotics (MIX). The data is represented as means ± SEM. The evolution of tail SBP was analysed by two-way ANOVA with the Tukey’s multiple comparison test. The rest of the variables were tested with one-way ANOVA and Tukey post hoc test (morphological variables, and plasma anti-ds-DNA), or Kruskal–Wallis with Dunn’s multiple comparison (proteinuria, anti-cardiolipin, plasma IFNα). * *p* < 0.05, ^†^ *p* < 0.01, ^‡^ *p* < 0.001 in comparison with CTR; * *p* < 0.05, ^†^ *p* < 0.01, ^‡^ *p* < 0.001 in comparison with IMQ.

Topical IMQ, a TLR7 agonist, was used to induce lupus-like disease in mice not genetically prone to excessive TLR7 signalling, it was administered topically three times a week in alternate days to wild-type BALB/c mice [9]. IMQ exposition showed a high incidence of mortality [9,13]. The mouse mortality rate in each group treated with IMQ was the following: IMQ group, 25%, 2 dead mice out of 8; IMQ-VANCO group, 0%; and IMQ-MIX group, 12.5%, 1 dead mouse out of 8. As expected, IMQ-treated mice showed a progressive raise in systolic blood pressure (SBP) (Figure 1A), being approximately 35 mmHg higher in IMQ-treated animals than in CTR animals, at the experimental endpoint. No significant changes in heart rate were detected in the IMQ group (530.3 ± 23.9 bpm vs. 540.7 ± 20.3 bpm, CTR and IMQ groups, respectively).

The IMQ-induced lupus model is characterized by kidney injury linked to autoimmunity [9,13,22]. We determined the renal function by measuring proteinuria and kidney histopathology. We found significant higher protein concentration in urine (Figure 1B), moderate chronic perivascular and tubule-interstitial inflammatory infiltrate, mild hyaline casts in renal tubules, mild glomerular injury with extra-capillary crescent, and scan immunocomplex deposits in glomerular tuft in kidney from IMQ group (Figure S3), showing impaired renal function. In addition, increased renal hypertrophy, hepatomegaly, and splenomegaly (Figure 1C) and plasma levels of anti-dsDNA and anti-cardiolipin autoantibodies, and IFNα (Figure 1D) were found in IMQ mice. To assess the role of the gut microbiota, we reduced the bacterial mass using vancomycin (VANCO) and broad-spectrum antibiotics (MIX). VANCO and MIX showed a reduction in total DNA levels in the colonic content by ≈73% and ≈89%, respectively (Figure S4). As described previously [22], MIX treatment improved renal physiology by decreasing proteinuria (Figure 1B) and the inflammatory infiltrate and the glomerular injury in the kidney (Figure S3), it decreased splenomegaly, hepatomegaly, and renal hypertrophy (Figure 1C), and plasma levels of anti-dsDNA, anti-cardiolipin, and IFNα (Figure 1D), but did not inhibit the development of hypertension in IMQ-treated mice (Figure 1A). However, VANCO treatment prevented autoimmunity (reduced plasma levels of anti-dsDNA, anti-cardiolipin, and IFNα) (Figure 1D), proteinuria (Figure 1B), and renal injury histological features (Figure S3), but also reduced the progressive increase in SBP induced by IMQ, by approximately −15 mmHg (Figure 1A). The effects of vancomycin in SBP were independent of water consumption since mean water intake during treatments was similar among all experimental groups (mL mouse^−1^ day^−1^: CTR, 4.1 ± 0.2; IMQ, 4.3 ± 0.1; VANCO 4.2 ± 0.2; MIX 3.8 ± 0.3). Interestingly, when control mice were treated chronically with vancomycin or MIX, without IMQ, no significant changes in SBP (Figure S5A) and proteinuria (Figure S5B) were observed as compared to untreated control group, showing specificity under conditions of TLR7 activation. It also interesting to note the heterogeneity of some individual responses specific to the IMQ group observed in one or two animals. The reasons for this are unknown, but could be related to the variability in the topical administration of this drug.

These data point to the gut microbiota being needed for TLR7-dependent systemic autoimmunity, but only vancomycin-sensitive gut microbiota was involved in the hypertensive effect induced by TLR7 activation. We proceeded to study the gut microbial community composition derived from both antibiotic treatments, to assess potential pathobionts regulating TLR7-dependent hypertension.

### 3.2. Antibiotic Treatment Changed Colonic Microbiota Composition

**Figure 2:**
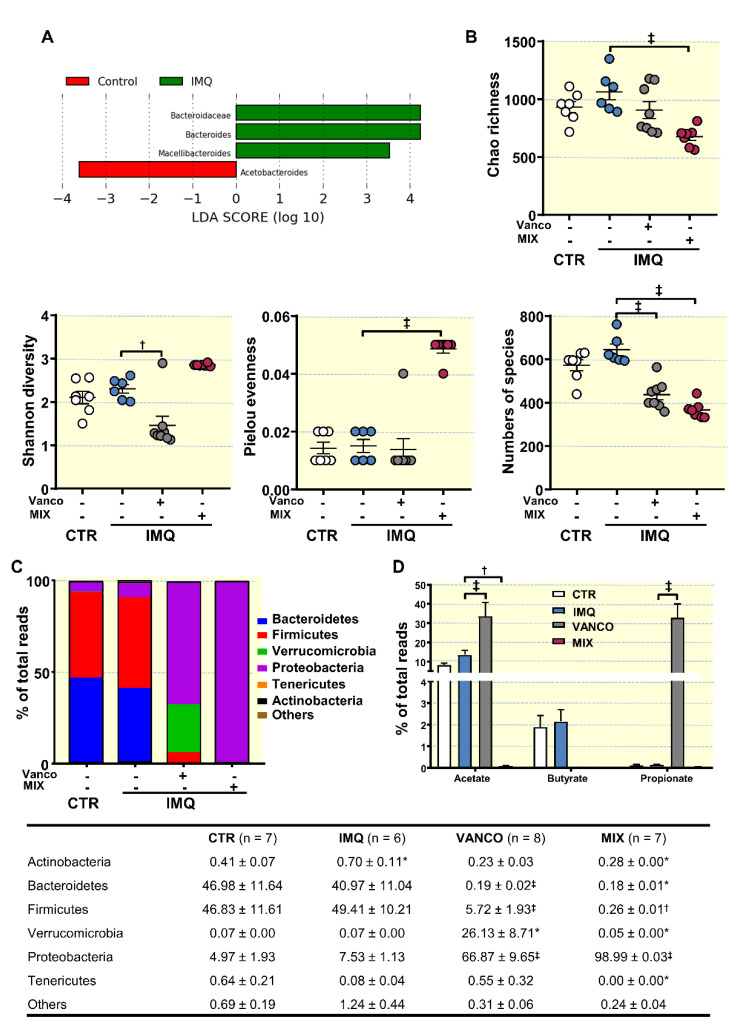
Effects of treatment with antibiotics on ecological indices and microbial phyla of in the gut from imiquimod (IMQ) mice. (**A**) Linear discriminant analysis Effect Size (LEfSe) recognized significant differences in bacterial taxa enriched in each cohort at LDA Score > 3.5, and Kruskal–Wallis test amongst classes and Wilcoxon test amongst subclasses with threshold 0.05 (red bars Control enriched, green bars IMQ enriched). (**B**) Ecological parameters, (**C**) Proportion of bacterial phyla, and (**D**) Proportion of SCFAs-producing-bacteria in the microbiota from control (CTR), IMQ and IMQ-groups treated with Vancomycin (VANCO) or our cocktail of antibiotics (MIX). Data is represented as means ± SEM. One-way ANOVA and Tukey post hoc test (ecological parameters, and phyla proportion), two-way ANOVA with the Tukey’s multiple comparison test (SCFA-producing bacteria proportion). * *p* < 0.05 compared to CTR group; * *p* < 0.05, ^†^ *p* < 0.01, ^‡^ *p* < 0.001 compared to the untreated IMQ group.

We collected colonic content samples from mice, isolated bacterial DNA, and performed high-throughput 16S ribosomal DNA sequencing. The bacterial taxa (class, order, family, and genus) that experienced changes in IMQ mice, as indicated in our Linear Discriminant Analysis (LDA), exposed that the relative abundance of 3 taxa was increased (green) and 1 taxon was decreased (red) as compared to CTR group (Figure 2A). No significant differences in the ecological parameters (Chao richness, Shannon diversity, Pielou evenness and numbers of species) were found between both groups (Figure 2B). However, increased bacteria belonging to Actinobacteria phylum was detected in IMQ group (Figure 2C). Antibiotic treatments induced profound changes in taxa as compared to IMQ group, according to the LDA (Figure S6). Vancomycin is a drug that mainly eradicates Gram-positive microorganisms and cannot be absorbed in the intestines. Similar to that described in vancomycin-treated female lupus prone MRL/lpr mice [47], vancomycin treatment of IMQ mice reduced Shannon diversity (the combined parameter of richness and evenness) and numbers of species, while MIX showed a reduction in richness (an estimate of a total number of OTUs present in the given community), and numbers of species, but increased evenness (that shows how uniformly individuals in the community are distributed over different OTUs) (Figure 2B).

Very significant shifts in phyla proportions were detected, especially in MIX (Figure 2C). The ratio between Bacteroidetes and Firmicutes was decreased whereas Verrucomicrobia and Proteobacteria were higher in VANCO as compared to IMQ, similar to that previously described with vancomycin treatment in high fat diet-fed mice [48] and in MRL/lpr mice [47]. In agreement with previous report using the same mixture of broad-spectrum antibiotics in young and old mice [49], Proteobacteria was the most abundant phylum in the microbiota from MIX group (≈99%), with a profound depletion of Bacteroidetes and Firmicutes. Within the phylum Firmicutes, vancomycin induced the decline of class Clostridia, without significant changes in *Erysipelotrichia* and *Bacilli* (not shown). Within the class Clostridia, every major family decreased, including *Clostridales* and *Lachnospiraceae* (not shown). This was accompanied by a significant increase of Gram negative Proteobacteria family *Enterobacteriaceae* (not shown). At genus level, the proportion of *Barnesiella* (Bacteroidetes, *Porphyromonadaceae*) and *Clostridium XIVa* (*Clostriales*) was reduced and *Escherichia/Shigella* (*Enterobacteriaceae*) was increased by both antibiotic treatments (Figure S5). By contrast, the proportion of Gram-negative bacteria *Akkermansia* (Verrucomicrobia) and *Parasutterella* (Proteobacteria) was increased by vancomycin but not by MIX treatment (Figure S7). Zegarra-Ruiz et al., [22], using 16S rDNA sequencing of faecal samples from TLR7-dependent mouse models of SLE and from SLE patients compared with healthy controls, showed Gram-positive bacteria *Lactobacillus* ssp. (*Bacilli*) enrichment and translocation to internal organs. However, in our experimental conditions, the relative populations of *Lactobacillus* were not significatively different between CTR and IMQ group, which were unaffected by vancomycin (consistent with the known resistance of several species of lactobacilli to vancomycin) and reduced by MIX.

SCFAs are metabolites resulting from the fermentation of undigested carbohydrates in gut microbiota with known beneficial effects [50]. However, no significant changes in the proportion of SCFAs-producing bacteria were found between CTR and IMQ groups (Figure 2D). Interestingly, vancomycin treatment increased acetate- and propionate-producing bacteria (Figure 2D). SCFAs could exert certain actions on intestinal cells and on at local immune system level. We next investigated whether antibiotic treatments induced changes in immune cells in secondary lymph organs.

### 3.3. Antibiotic Treatments Attenuates T Cells Imbalance

An elevated production of autoantibodies and the progression of the lupus-like autoimmune pathology can be associated with an imbalance of T cells [51] and B cell proliferation [13,52]. We assessed B and T cell levels in mesenteric lymph nodes (MLNs) and spleens from all experimental groups.

**Figure 3:**
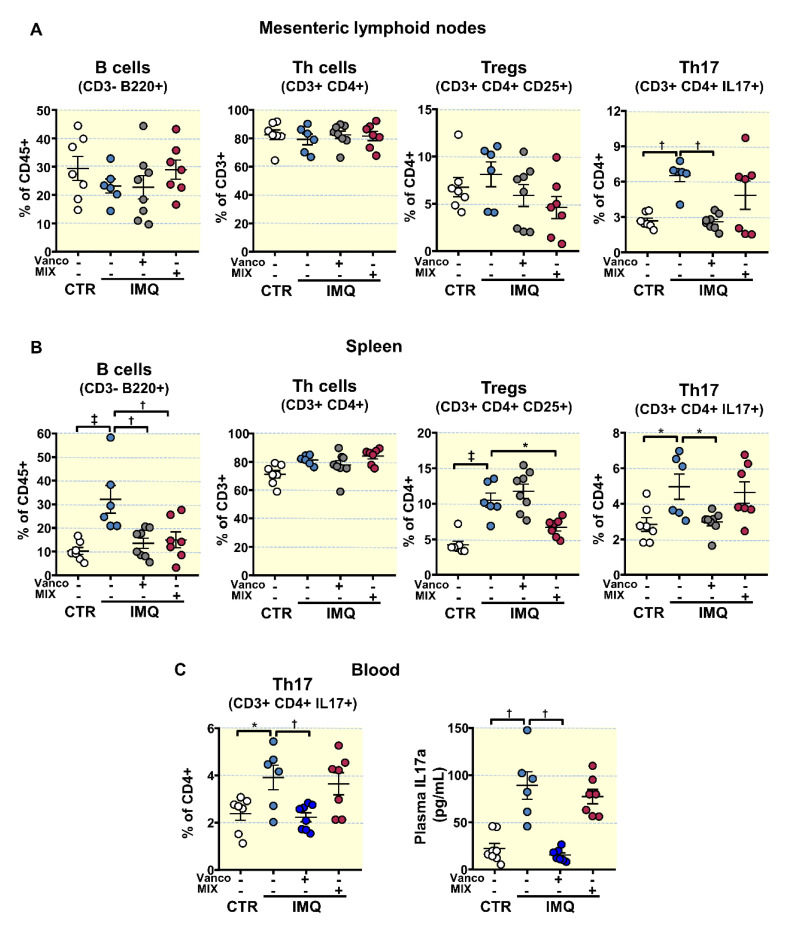
Effects of treatment with antibiotics on lymphocyte activation and proliferation in imiquimod (IMQ) animals. (**A**) Total B lymphocytes, T helper (Th), Regulatory T (Treg), and Th17 cells as detected with flow cytometry in mesenteric lymph nodes, (**B**) in spleen, and (**C**) blood Th17 cells and plasma IL17a levels from control (CTR), IMQ and IMQ concomitantly treated with Vancomycin (VANCO) or our cocktail of antibiotics (MIX). Data is represented as means ± SEM. The variables were tested with one-way ANOVA and Tukey post hoc test. Plasma IL17a levels were compared using Kruskal–Wallis with Dunn’s multiple comparisons test. * *p* < 0.05, ^†^ *p* < 0.01, ^‡^ *p* < 0.001 compared to the CTR group; * *p* < 0.05, ^†^ *p* < 0.01 compared to the untreated IMQ group.

B cell relative populations (CD3-B220+) were higher in spleen from IMQ mice than in the CTR group, but not in MLNs (Figure 3A,B). VANCO and MIX treatments reduced splenic B cell populations (Figure 3B). The proportion of T helper (Th) cells (CD3+CD4+) presented no significant differences among all experimental groups in both MLNs and spleen. T regulatory (Treg, CD4+CD25+) and Th17 (CD4+IL-17a+) cell relative populations increased in spleen from IMQ mice (Figure 3B), whereas only Th17 cells where higher in lupus disease in the MLNs (Figure 3A). VANCO treatment prevented the raise in Th17 cell content as seen in IMQ in both secondary lymph organs, being without effect in the proportion of Treg cells. In contrast, MIX treatment did not change Th17 cell population but reduced Treg content in the spleen (Figure 3A,B).

Circulating Th17 lymphocytes were increased in IMQ group compared to control mice (Figure 3C). In line with changes brought by treatment with the antibiotics in MLNs, VANCO decreased circulating Th17 cell populations, being without the effects of MIX. Plasma levels of IL-17a were higher in IMQ than in CTR (Figure 3C), which were reduced by vancomycin treatment. Taken account that IL-17a is a key factor contributing to endothelial dysfunction in this TLR7-driven lupus autoimmunity model [13], we next examined the changes brought by the antibiotics on the observed SLE-linked endothelial dysfunction.

### 3.4. The Antibiotics Prevented Endothelial Dysfunction, Vascular Oxidative Stress and Th17 Vascular Infiltration

**Figure 4:**
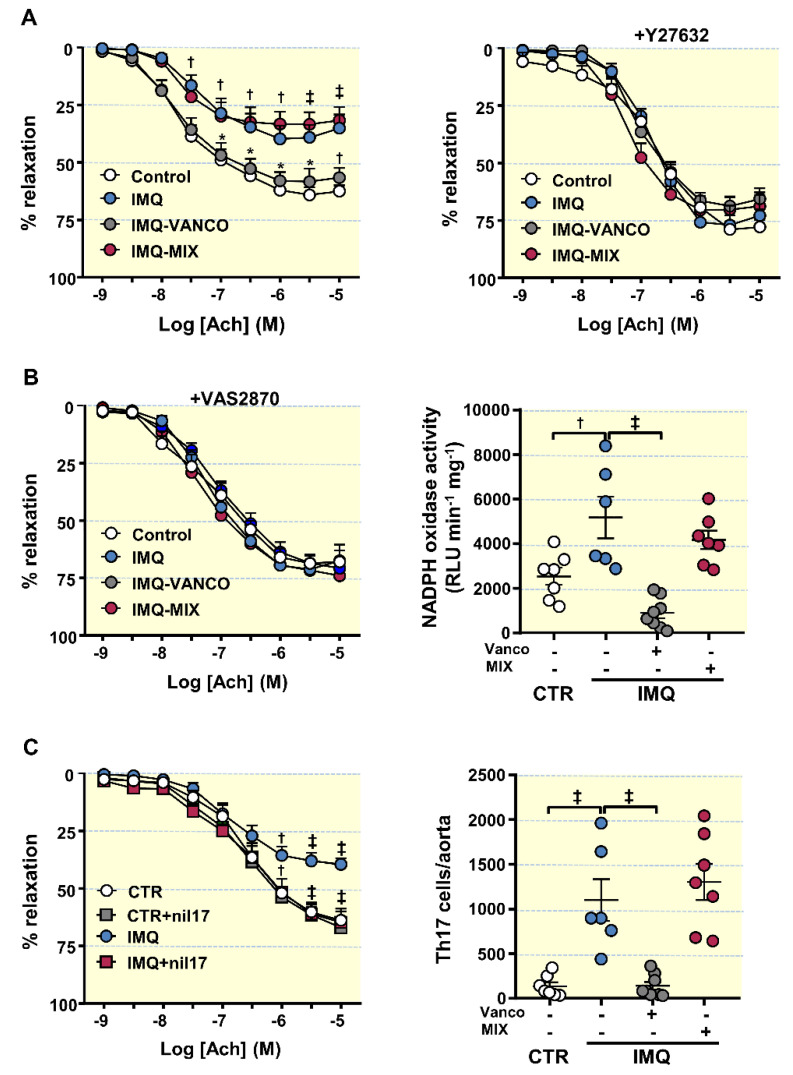
Effects of treatment with antibiotics on SLE-linked endothelial dysfunction, NADPH oxidase activity and immune cell aorta infiltration in imiquimod (IMQ) mice. (**A**) Acetylcholine (Ach)-induced relaxation responses in aortas pre-contracted by U46619 (10 nM), with or without the Rho kinase inhibitor Y27632 (1 µM); control (CTR), IMQ and IMQ-groups treated with Vancomycin (VANCO) or our cocktail of antibiotics (MIX). (**B**) Aortic responses induced by Ach in the absence or in the presence of the specific pan-NOX inhibitor VAS2870 (1 µM) and aortic NADPH oxidase activity determined using a lucigenin-enhanced chemiluminescence. (**C**) Aortic responses induced by Ach in the absence or in the presence of anti-IL-17a antibody (10 µg·L) and aortic infiltration of Th17 assessed with flow cytometry. Data is represented as means ± SEM. The concentration-response curves to Ach were analysed by two-way ANOVA with the Tukey’s multiple comparison test. The rest of the variables were tested with one-way ANOVA and Tukey post hoc test. ^†^ *p* < 0.01, ^‡^ *p* < 0.001 in comparison with CTR; * *p* < 0.05, ^†^ *p* < 0.01, ^‡^ *p* < 0.001 in comparison with IMQ.

IMQ-treated animals presented strongly reduced endothelium-dependent vasorelation to ACh (acetylcholine) in comparison CTR animals (Emax = 39.7 ± 6.2% and 64.1 ± 3.6%, respectively, *p* < 0.01) (Figure 4A). VANCO was able to improve this impairment, being without effect MIX. The ACh-induced response was also improved in aorta segments from IMQ-treated animals post-Y27632 incubation (a Rho kinase inhibitor) (Figure 4A), suggesting that the decreased relaxation with ACh is mediated, at least partially, by Rho kinase activation. The activation of RhoA/Rho kinase by ROS has already been previously described [53]. NADPH oxidase is the main source of ROS in the vascular wall; thus, we tested the relaxation to ACh in the presence of the selective NADPH oxidase inhibitor VAS2870, and the NADPH oxidase activity in all experimental groups (Figure 4B). In the presence of VAS2870, improvement of endothelium-dependent relaxation to acetylcholine was observed in aortic rings from IMQ group, being similar to that found in CTR group, which suggest the involvement of NADPH oxidase activity in the endothelial dysfunction found in aortic rings from IMQ mice. In fact, the NADPH oxidase activity was approximately 2-fold more elevated in segments of IMQ mice than in aortic segments of CTR animals. In agreement with this, MIX treatment, which was unable to improve the relaxation to acetylcholine, showed an NADPH oxidase activity similar IMQ rings, whereas rings from VANCO group showed lower NADPH oxidase activity than IMQ group. To further explore the role of IL-17a in endothelial dysfunction induced by IMQ, we incubated for 6 h with anti-IL-17a antibody. The neutralization on IL-17a improved the relaxation to acetylcholine in rings from IMQ group, but not in control rings (Figure 4C). Considering that Th17 cells boosted vascular ROS synthesis, we determined aortic infiltration levels of Th17 lymphocytes. Th17 infiltration was higher in aorta from IMQ than in CTR, which was reduced by vancomycin but not by MIX treatment (Figure 4C). Interestingly, when control mice were treated with VANCO or MIX no significant changes were observed in the relaxation to acetylcholine (Figure S5C) or in the NADPH oxidase activity (Figure S5D), as compared to untreated control mice.

Hierarchical cluster analysis (according distance measure using Pearson test correlation and clustering algorithm using ward.D) represented as a heat map of the fifty most abundant bacterial genera detected among groups, showed clear separation of groups to cluster by BP and Th17 population in MLNs (Figure S8). We subsequently examined whether the enriched gut bacterial communities partake in Th17 polarization in MLNs and high BP-associated with TLR-7 activation.

### 3.5. Bacterial Communities from IMQ-Mice Were Transferable and Induced High Blood Pressure and Endothelial Dysfunction in Control Animals

**Figure 5:**
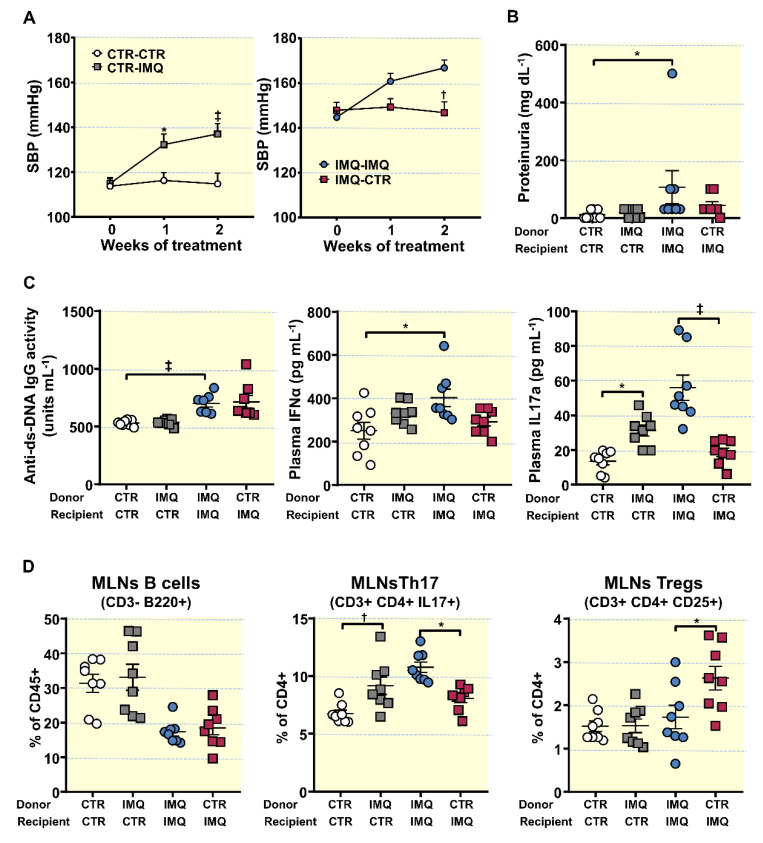
Effects of faecal microbiota transplants (FMT) on the progression of systolic blood pressure (SBP), proteinuria, disease activity, plasma cytokine levels, and lymphocyte proliferation in mesenteric lymph nodes. (**A**) SBP determined via tail-cuff plethysmography. (**B**) Proteinuria, (**C**) Plasma double-stranded DNA (anti-ds-DNA) autoantibodies, interferon (IFN)α, and interleukin (IL)17a levels. (**D**) Populations of relevant lymphocytes in mesenteric lymph nodes: Total B lymphocytes, Th17, and Regulatory T cells (Treg), obtained with flow cytometry. Data is represented as means ± SEM. The evolution of tail SBP was analysed by two-way ANOVA with the Tukey’s multiple comparison test. The rest of variables by unpaired t-test. * *p* < 0.05, ^†^ *p* < 0.01, ^‡^ *p* < 0.001 compared to the CTR-CTR group; * *p* < 0.05, ^†^ *p* < 0.01, ^‡^ *p* < 0.001 compared to IMQ-IMQ group. CTR-CTR, Control mice (CTR) transplanted with microbiota from CTR; CTR-IMQ, CTR mice transplanted with microbiota from imiquimod (IMQ)-treated mice; IMQ-IMQ, IMQ transplanted with microbiota from IMQ; and IMQ-CTR, IMQ transplanted with microbiota from CTR.

To address the question whether microbiota from lupus mice induced by TLR7 activation might affect BP and endothelial function regulation, we transplanted microbiota for 2 weeks from hypertensive IMQ or from normotensive CTR animals to recipient BALB/c female mice or 8 weeks IMQ-treated mice (Figure S9). Faecal microbiota transplants (FMT) from donor IMQ microbiota to recipient control mice increased SBP to a maximum of ≈22 mmHg, being without effect FMT from donor control microbiota to recipient control mice (Figure 5A). However, no significant change either in proteinuria (Figure 5B), plasma levels of anti-dsDNA and IFNα (Figure 5C), or B cells and Treg population in MLNs (Figure 5D) were detected between CTR-CTR and CTR-IMQ, displaying no changes in lupus activity induced by transplant from IMQ mice. Interestingly, this microbiota increased plasma IL17 levels (Figure 5C), and the Th17 proportion in MLNs (Figure 5D).

**Figure 6:**
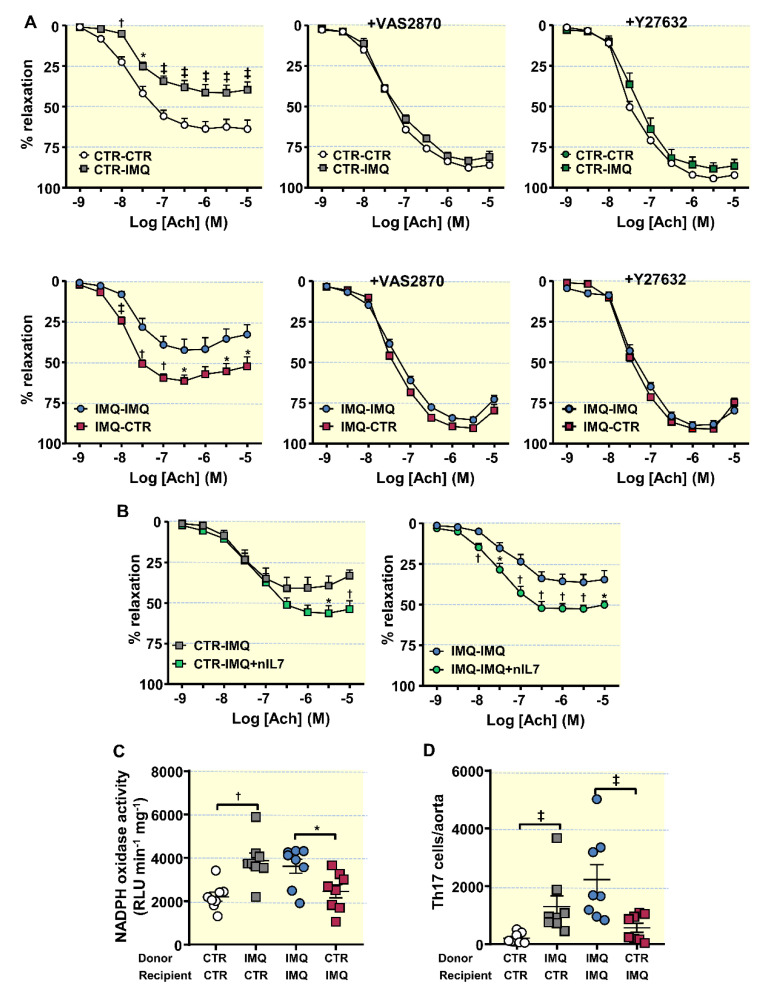
Effects of the microbiota transplants on endothelial function, NADPH oxidase activity and infiltration of T lymphocytes in aorta. (**A**) Acetylcholine (Ach)-induced relaxation curves in aortas pre-contracted with U46619 (10 nM) with or without the specific pan-NOX inhibitor VAS2870 (1 µM), or the Rho kinase inhibitor Y27632 (1 µM). (**B**) Acetylcholine (Ach)-induced relaxation curves with or without anti-IL-17a antibody (10 µg·L) in segments from control (CTR) and Imiquimod-treated (IMQ) recipient mice, transplanted with microbiota from IMQ animals. (**C**) NADPH oxidase activity determined via lucigenin-enhanced chemiluminescence. (**D**) Aortic infiltration of T helper(h)17 through flow cytometry. Values are represented as means ± SEM. The concentration-response curves to Ach were analysed by two-way ANOVA with the Tukey’s multiple comparison test. The rest of the variables were tested with unpaired *t*-test. * *p* < 0.05, ^†^ *p* < 0.01, ^‡^ *p* < 0.001 compared to the CTR-CTR group; * *p* < 0.05, ^†^ *p* < 0.01, ^‡^ *p* < 0.001 compared to IMQ-IMQ group CTR-CTR, Control mice (CTR) transplanted with microbiota from CTR; CTR-IMQ, CTR transplanted with microbiota from imiquimod (IMQ)-treated mice; IMQ-IMQ, IMQ transplanted with microbiota from IMQ; and IMQ-CTR, IMQ transplanted with microbiota from CTR.

Additionally, Ach-induced endothelium-dependent relaxation in U46619-contracted segments of aorta from CTR-IMQ were decreased when compared to CTR-CTR group (Emax: 41.5 ± 4.9% vs. 63.7 ± 4.8%, *p* < 0.01, respectively; Figure 6A).

Incubation for 30 min with the pan-NOX inhibitor VAS2870 or with the Rho kinase inhibitor Y27632 depleted the differences between groups in relaxation to acetylcholine, suggesting a role for NADPH oxidase and Rho kinase, respectively, in this impaired relaxant response induced by IMQ microbiota. Furthermore, the transplant from IMQ raised NADPH oxidase activity in aorta (Figure 6C), as compared to CTR-CTR. Remarkably, nIL-17 incubation increased relaxation to acetylcholine in rings from CTR-IMQ group (Figure 6A), showing the role of IL17 in the impaired endothelium-dependent relaxation induced by stool transplantation with IMQ microbiota. In fact, Th17 infiltration in aorta was also higher in CTR-IMQ than in CTR-CTR (Figure 6D). By contrast, FMT from control mice to recipient IMQ-treated mice reduced SBP (≈ −20 mmHg) (Figure 5A), Th17 proportion in MLNs (Figure 5C), plasma IL-17 levels (Figure 5B), aortic NADPH oxidase activity (Figure 6C), aortic Th17 cells infiltration (Figure 6D), and the impaired acetylcholine-induced relaxation as compared to IMQ-IMQ group (Figure 6A), confirming the key role of IL-17 in the vascular alteration induced by IMQ. In fact, neutralization of IL-17 improved endothelium-dependent relaxation in IMQ-IMQ group (Figure 6B) similarly to stool transplantation from control mice. However, no significant changes in proteinuria (Figure 5B), and plasma anti-dsDNA and IFNα (Figure 5C) were found between IMQ-IMQ and IMQ-CTR groups, showing a dissociation between the control of autoimmunity and SBP induced by the gut microbiota.

**Figure 7:**
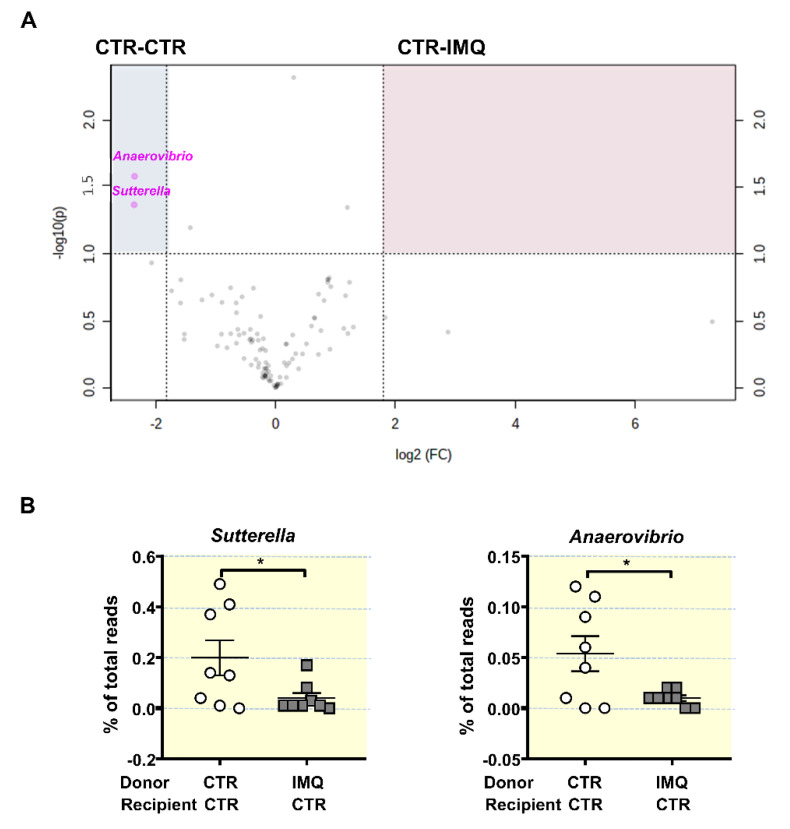
Effects of stool transplantation from imiquimod (IMQ)-treated mice to control mice at the genera proportion in gut microbiota. (**A**) Volcano plot displaying significantly enriched genera in CTR-CTR group or CTR-IMQ group. The x-axis is log2 fold change and the y-axis is transformed *p*-value. Genera not different between cohorts are shown in black. Features selected by volcano plot with fold change threshold 3.5 and *t*-test with threshold 0.05. Genera more abundant in CTR-CTR are shown in the upper right, blue-shaded quadrant (**B**) Proportion of key bacterial genera expressed as percentage of total reads. Data is represented as means ± SEM. Unpaired *t*-test. * *p* < 0.05 compared to the CTR-CTR group. CTR-CTR, Control mice (CTR) transplanted with microbiota from CTR; CTR-IMQ, CTR transplanted with microbiota from imiquimod (IMQ)-treated mice.

Next, tested the microbial composition 2 weeks post-transplant. Microbiota from donor CTR and IMQ mice changes after FMT in recipient mice (Figure S9A), but with minor changes depending if recipient mice were CTR or IMQ mice. At the end of FMT donor microbiota from control mice showed increased bacteria belonging to Bacterioidetes (75.18 ± 3.43% vs. 55.06 ± 8.55%, *p* < 0.05) and reduced Actinobacteria (0.09 ± 0.01% vs. 0.15 ± 0.03%, *p* < 0.05) in recipient IMQ mice in comparison with recipient CTR mice, respectively. Similarly, donor microbiota from IMQ mice showed only reduced bacteria belonging to Tenericutes (0.08 ± 0.02% vs. 0.25 ± 0.04%, *p* < 0.01) in recipient IMQ mice as compared to recipient CTR mice. We found no significant changes in phyla composition between CTR-CTR and CTR-IMQ groups (Figure S9B). Volcano plot showed genera clustering in CTR-CTR mice as compared to CTR-IMQ group (Figure 7A). There was more than 3.5-fold reduction in the genera *Sutterella* and *Anaerovibrio* (potentially protective bacteria) in mice receiving IMQ microbiota in comparison with CTR-CTR mice (Figure 7B). By contrast, FMT of donor control faeces to IMQ-treated mice induced changes in the microbiota composition as compared to the transplantation with IMQ microbiota. The LDA score suggests that the relative abundance of 4 taxa (green) was decreased and 15 taxa (red) was increased in IMQ-CTR group when compared with IMQ-IMQ mice (Figure S10). The most altered genera abundances of the volcano plot showed increased proportion of *Sutterella, Anaerovibrio*, *Heliorestis*, *Sharpea*, *Anaerobranca*, and *Halanaerobium*, and reduced abundance of *Pseudovutyrivibrio*, *Moryella*, and *Streptococcus* in IMQ-treated mice transplanted with microbiota from control mice, as compared to IMQ-IMQ group (Figure S11A,B).

## 4. Discussion

With the present experiments, we have demonstrated the relevance of gut microbiota as a regulating agent for endothelial function and BP in a systemic autoimmunity model induced by TLR-7 activation, which mimicked SLE patients with high IFN signature (>80% patients). This is especially suggested by the observed changes in endothelial dysfunction, vascular oxidative stress, and SBP induced by shifts in gut microbiota composition after both chronic vancomycin treatment and stool transplantation of microbiota from control mice to IMQ-treated mice. The beneficial effects of both interventions were associated with reduced polarization of Th cells to Th17 in MLNs, with the subsequent reduced Th17 cells/IL17a in blood and reduced Th17 infiltration in the aorta. It is interesting to note that a profound decrease in colonic biomass in MIX mice, reduced autoimmunity, improved kidney function, as described previously using two different TLR7-dependent lupus models [22], but did not prevent endothelial dysfunction and the raise of SBP, showing a clear dissociation between autoimmunity and BP. In addition, MIX treatment was unable to reduce Th17 polarization in secondary lymph organs and Th17 infiltration in aorta, confirming the key role of IL17 in the vascular alterations induced by TLR-7 activation. IL-17A has also been implicated as a pathogenicity factor in a number of chronic inflammatory diseases, including multiple sclerosis, arthritis, and psoriasis. However, our present data are in agreement with that showing that IL-17 are not a direct mediator of autoimmunity [54], but it controls microbiota-mediated vascular dysfunction induced by TLR7-activation.

Profiling colonic microbiomes from TLR7-dependent lupus mice showed an enrichment of mainly three bacterial taxa: the genera *Bacteroides* and *Macellibacteroides*, and the family *Bacteroidaceae*, whereas *Acetobacteroides* was enriched in CTR group. However, these genera were reduced by MIX treatment, which was unable to reduce BP, suggesting that they do not contribute to increase BP in IMQ-treated mice. Dysbiosis in hypertension is mainly associated with reduced proportions of SCFAs-producing bacteria, mainly acetate- and butyrate-producing bacteria [55]. However, no significant changes in SCFAs-producing bacteria were found between CTR and IMQ group suggesting that these SCFAs are not involved in the development of high BP. Interestingly, vancomycin treatment increased acetate- and propionate-producing bacteria associated with BP reduction, whereas MIX treatment was without effect in the proportion of SCFAs-producing bacteria. These data suggest that regulating bacterial SCFAs production, such as increasing acetate and propionate, could be involved in BP regulation in IMQ-treated mice. In fact, we demonstrated recently that chronic oral acetate consumption could prevent the increase in blood pressure in spontaneously hypertensive rats, reducing Th17 population in MLNs, and restoring Th17/Treg balance in aorta [50]. Similarly, propionate attenuated vascular dysfunction and hypertension by increasing splenic Treg cells and reducing Th17 cells [56]. Unfortunately, we did not measure plasma levels of SCFAs. Taken into account that SCFAs have direct vasculoprotective effects in vascular wall [57], we cannot exclude whether lower plasma SCFAs levels in the IMQ group are involved in high BP, and if vancomycin improved endothelial function and reduced BP by increasing plasma acetate or propionate concentrations.

Vancomycin, but not MIX, treatment enriched *Akkermansia* and *Parasutterella. A. muciniphila,* which was 53.56% of all species found in gut microbiota from VANCO group, induces intestinal adaptive immune responses during homeostasis. T cell responses to *A. muciniphila* appear to be context dependent [58]. In fact, *A. muciniphila*, which was increased in patients with multiple sclerosis [59], induced proinflammatory responses in human peripheral blood mononuclear cells and in monocolonized mice [60]. However, this microorganism improves the metabolism of obese and diabetic mice [61], and immune system in hyperlipidaemic E3L.CETP mice [62]. In our experiment, in the setting of TLR7-activation, *A. muciniphila* is associated with reduced Th17 cells in MLNs and spleen. Among the potential mechanisms, it has been shown that *A. muciniphila* expresses specific proteins on its outer membrane, such as the protein Amuc_1100. This protein binds and activates TLR2, which potently inhibits TLR4- and TLR7/8-induced cytokine production by dendritic cells [63]. Moreover, enrichment of *Parasutterella* has beneficial effects in immune function [64]. Most notably, when transplanted to a control mouse, microbiota from IMQ mice during 2 weeks induced a significantly increase in SBP and reduced endothelium-dependent relaxation to acetylcholine, without change in autoimmunity and proteinuria as compared to the healthy control microbiota transplantation. Our data implies that the modified microbiota has a crucial part in the onset and progression of hypertension and is one of the triggering elements for SLE hypertension, instead of being a result and accompanying phenomenon in the development of lupus after TLR7 activation. In addition, vancomycin treatment reduced BP, whereas a more aggressive microbiota depletion using a broad-spectrum antibiotic mixture, which also contained a lower dose of vancomycin, was unable to change the development of hypertension in IMQ mice. This is consistent with the finding that the interaction between the gut microbiome and BP is very complex and depends on multiple host factors as well as the environment.

The microbial profiles of the colonized mice showed several differences, including lower *Sutterella*, an organism shown to induce a protective immunoregulatory profile in vitro [59], and *Anaerovibrio* proportion. These microbial changes were also associated with increased Th17 proportions in MLNs, plasma IL-17a levels, and Th17 infiltration in aorta in control mice transplanted with IMQ microbiota. Interestingly, control stool transplantation to IMQ-treated mice enriched *Sutterella* and *Anaerovibrio* in faeces, associated with reduced Th17 polarization and improvement of endothelial function and hypertension, suggesting a role of these bacteria in BP control in this model of autoimmunity.

Several studies have shown that multiple elements partake to the onset of SLE hypertension, including the inflammatory cytokines, and oxidative stress, as well as B-cell hyperactivity and autoantibody production [5]. These elements, that mediate in local inflammation and the subsequent renal and vascular dysfunction, are likely downstream of the initial immune system dysregulation [65]. In line with these findings, we detected high plasma concentrations of pro-inflammatory cytokines (IL-17a and IFNα), in IMQ mice, besides vascular oxidative stress, and increased plasma anti-ds-DNA. Remarkedly, interventions reducing plasma IL17a levels, such as, vancomycin treatment and transplantation of control microbiota, reduced high BP induced by TLR7 activation. In addition, FMT from IMQ group to control mice increased plasma IL-17, suggesting the key role of IL17a as a mediator in the pro-hypertensive impact of microbiota in IMQ mice. These data are similar to that described after FMT transplantation from NZBWF1 mice, which composition is quite different to IMQ group, to germ-free or germ-depleted mice [25], showing that the polarization of naïve T cells toward Th17 cells induced by several bacteria is a key event to increase BP. The involvement of B-cell hyperactivity and autoantibody synthesis as mediator of vascular changes brought by microbiota was ruled out, since MIX treatment reduced plasma autoantibody but did not prevent BP increase and faecal transplant from IMQ-treated animals to CTR mice raised BP but not anti-ds-DNA antibodies.

Hypertension is often associated with impaired endothelial function, but if this is causative in the progression of hypertension is difficult to prove. Recently, it has been shown impaired endothelium-dependent relaxation responses to acetylcholine in aortas from TLR7-activated mice [13,66]. High NADPH oxidase ROS synthesis can be linked to the loss of endothelial function and the increase in BP in female IMQ-treated animals [13]. Consistently, in these experiments we also detected a decreased acetylcholine-dependent relaxation and high NADPH oxidase activity in aortic rings from IMQ group in comparison with CTR. Curiously, VANCO or transplantation of healthy microbiota prevented both pathological effects, linking the microbiota to oxidative stress and endothelial function. In accordance, faecal transplant from IMQ to CTR had the opposite effect, impairing acetylcholine-induced vasorelaxation and increasing NADPH oxidase activity. NADPH oxidase ROS synthesis has a crucial part in endothelial dysfunction caused by microbiota since the selective NADPH oxidase inhibitor VAS2870 improved the vasorelaxation to acetylcholine induced by stool transplantation of donor IMQ.

Proinflammatory cytokines in plasma, or local production in the vascular tissues, due to infiltrating inflammatory cells, control NADPH oxidase activity [67,68]. Both vancomycin treatment and FMT from control mice decreased Th17 maturation and proliferation in MLNs, circulation, and infiltration in aorta. The pro-inflammatory cytokine IL-17 is known to cause a loss of endothelial function due to Rho kinase activation in the vascular wall [69], at least partially, by raising NADPH oxidase-generated ROS [70]. Moreover, the Rho-kinase inhibitor Y27632 improved the response to acetylcholine in CTR-IMQ group, which suggests that the IL-17-Rho kinase pathway is regulated by the microbiota from IMQ mice. Consistently, the faecal transplant from IMQ to CTR raised Th17 cell levels in MLNs, and its infiltration in aorta. Furthermore, nIL-17 reverted acetylcholine relaxation in CTR with IMQ microbiota to values similar to those of CTR-CTR. Overall, these data showed that regulation of naïve T cells maturation to Th17 in secondary lymphoid tissues at the gut, with the subsequent Th17 infiltration at vascular tissues, is a crucial component of subjacent causes behind the endothelial dysfunction induced by IMQ microbiota.

## 5. Conclusions and Limitations

These experiments show that: (1) there are differences between gut microbiota from hypertensive IMQ-treated mice and their appropriate controls, (2) this gut microbiota triggers changes in BP regulation, as proved by reduced BP induced by vancomycin treatment and stool transplantation from control mice, and (3) this can be linked to the activation of pro-inflammatory Th17 lymphocytes. Lupus is a female-biased disease with that affects females nearly 9:1 over males. Our data were obtained from female mice. Since proof exists supporting differences in gut microbiota composition between the sexes [71], the role of gut microbiota in BP regulation using male mice should be studied. Targeting the IL-17/IL-17receptor pathway may present an intriguing therapeutic strategy for Th17-induced hypertension in SLE patients. However, several adverse events for drugs blocking the IL-17 pathway, such as bacterial infections, mucocutaneous candidiasis, and neutropaenia have been reported. The present results open new possibilities to the prevention of SLE-associated cardiovascular complications by modulating of the gut microbiota composition, such as with the consumption of probiotics [26]. Nonetheless, caution should be taken when extrapolating these findings to humans due to the potential differences in the features of the animal and human gut microbiota. Moreover, the translation of the use of strategies of faecal transplantation performed in the present experimental model into the clinic, in example FTM from non-SLE human to SLE patients, needs further research, taken into account the specificity of the observed microbiota changes in this model, which were different than those observed in genetic SLE mouse [25], and possibly in human SLE patients. Despite FMT gaining considerable interest as a therapeutic approach in autoimmune diseases, their use in clinical practice may be limited due to practical objections in these chronic conditions. In addition, FMT from control to IMQ-treated mice reduced vascular alterations but did not reduce autoimmunity.

## Data Availability

The data presented in this study are available in this manuscript.

## Supplementary Materials

The following are available online at https://www.mdpi.com/article/10.3390/antiox10091426/s1, Figure S1: Experimental protocol for faecal microbiota transplantation (FMT), Figure S2: Gating strategy for flow cytometry, Figure S3: Effects of antibiotic treatments in renal injury in imiquimod (IMQ) mice, Figure S4: Effects of antibiotic treatments in DNA content of colonic microbiota in imiquimod (IMQ) mice, Figure S5: Effects of antibiotic treatments in blood pressure, proteinuria, and endothelial function in control (CTR) mice, Figure S6: Effects of antibiotic treatments in taxa of gut microbiota in imiquimod (IMQ) mice, Figure S7: Effects of antibiotic treatments in the main genera proportion of gut microbiota in imiquimod (IMQ) mice, Figure S8: Effects of antibiotic treatments in the main genera proportion of gut microbiota in imiquimod (IMQ) mice associated with systolic blood pressure (SBP) and Th17 proportion in mesenteric lymph nodes (MLN), Figure S9: Phyla proportion after faecal microbiota transplantation (FMT) experiment, Figure S10: Effects in taxa proportion of gut microbiota after donor control (CTR) microbiota transplantation to imiquimod (IMQ)-treated mice, Figure S11: Effects of stool transplantation from control mice to imiquimod (IMQ)-treated mice at the genera proportion in gut microbiota.
