# Supplementary material for: Gut Microbiota Has a Crucial Role in the Development of Hypertension and Vascular Dysfunction in Toll-like Receptor 7-Driven Lupus Autoimmunity
Source: Antioxidants (Basel). 2021 Sep 7;10(9):1426. doi: 10.3390/antiox10091426 (PMC8472682; doi:10.3390/antiox10091426)
Supplement: Supplementary file 1 [file antioxidants-10-01426-s001.zip › antioxidants-1346484-SI.pdf]

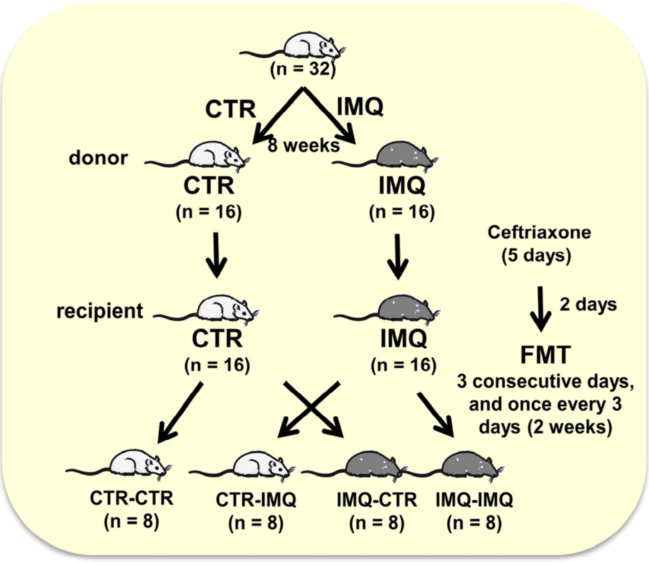

Figure S1. Experimental protocol for faecal microbiota transplantation (FMT). IMQ, imiquimod; CTR, control mice.

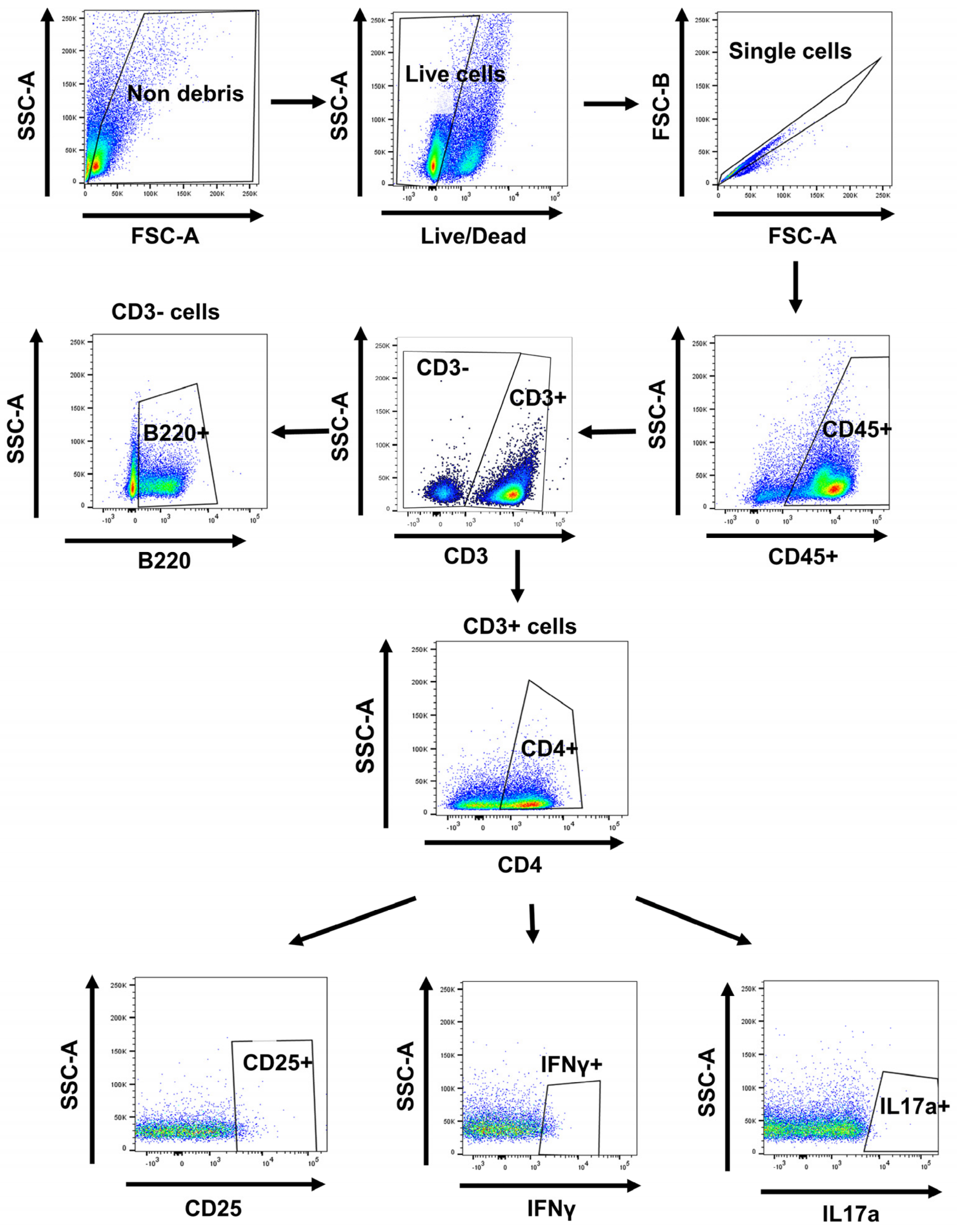

Figure S2. Gating strategy for flow cytometry.

A

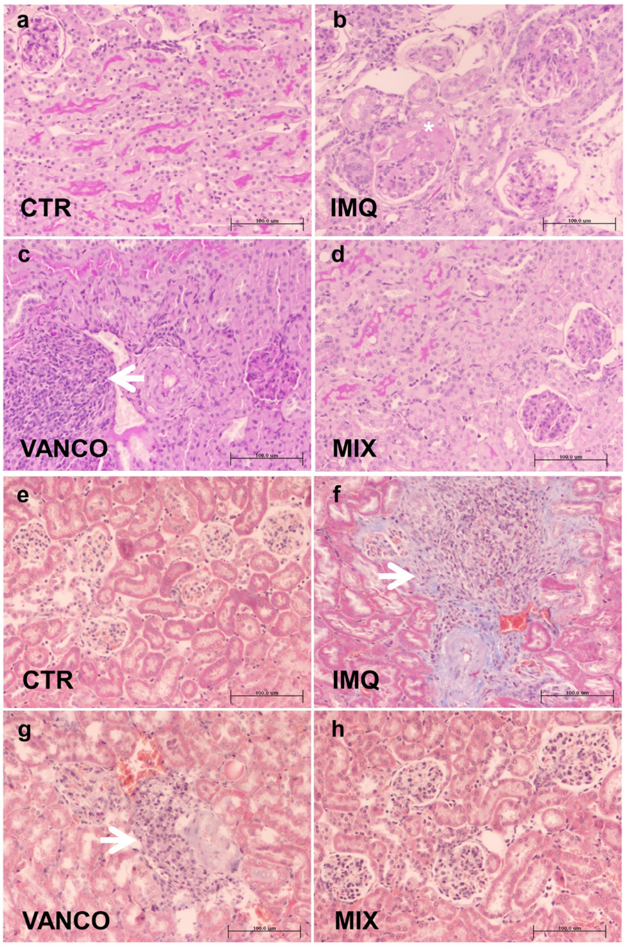

B

Variables	CTR (n = 7)	IMQ (n = 6)	VANCO (n = 8)	MIX (n = 7)
**Glomerulosclerosis (%)**	0.0±0.0 [0]	0.0±0.0 [0]	0.0±0.0 [0]	0.0±0.0 [0]
**Crescents (%)**	0.0±0.0 [0]	23.7±6.2† [80]	4.7±3.1* [37.5]	1.0±0.7† [20]
**Fuchsinophil deposits (%)**	0.0±0.0 [0]	0.60±0.27* [67]	0.0±0.0* [0]	0.0±0.0* [0]
**Mesangial sclerosis (%)**	0.0±0.0 [0]	0.0±0.0 [0]	0.0±0.0 [0]	0.0±0.0 [0]
**Cells/Glomerulus**	27.8±1,3	33.7±1.7*	33.1±1.5	27.6±1.5*
**Hyaline casts (0-3)**	0.4±0.1 [28]	1.0±0.0* [100]	0.9±0.1 [87]	0.1±0.1† [14]
**Inflammatory infiltrate (0-3)**	0.0±0.0 [0]	1.8±0.2† [100]	1.1±0.2* [100]	1.0±0.2* [100]

Figure S3. Effects of antibiotic treatments in renal injury in imiquimod (IMQ) mice. **(**A) Representative microphotographs of injury of mice renal cortex. Control group (CTR) with absence of tubulointerstitial lesions, preservation of the tubular brush border and normal glomerular size (a and e). IMQ group with glomerular size increase and presence of crescents (asterisk) and moderate perivascular chronic inflammatory infiltrate and mild fibrosis (arrow) (b and f). VANCO group with presence of mild perivascular chronic inflammatory infiltrate (arrow) (c and g). MIX group without tubulointerstitial or glomerular injury (d and h). (a to d, Periodic acid–Schiff [PAS] stain; f to I, Masson trichrome stain). Bar scale: 100 micrometers. (B) Table shows the quantification of renal lesions. Values are expressed as means ± SEM of percentage of affected glomeruli (n=50/mouse). The percentage of mice with lesion is expressed in brackets [mice %]. *P<0.05, †P<0.01 compared to the CTR group; *P<0.05, †P<0.01 compared to the untreated IMQ group.

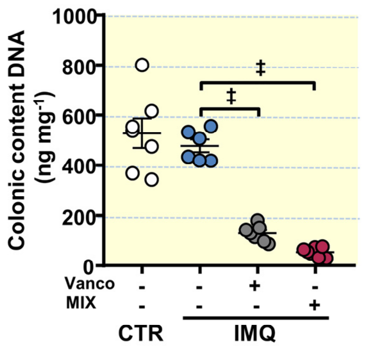

Figure S4. Effects of antibiotic treatments in DNA content of colonic microbiota in imiquimod (IMQ) mice. DNA content in the gut microbiota in control (CTR), IMQ and IMQ-groups treated with Vancomycin (VANCO) or a broad-spectrum antibiotic mixture (MIX). Values are expressed as means ± SEM. One-way ANOVA and Tukey *post hoc* test. ‡P<0.001 compared to the untreated IMQ group.

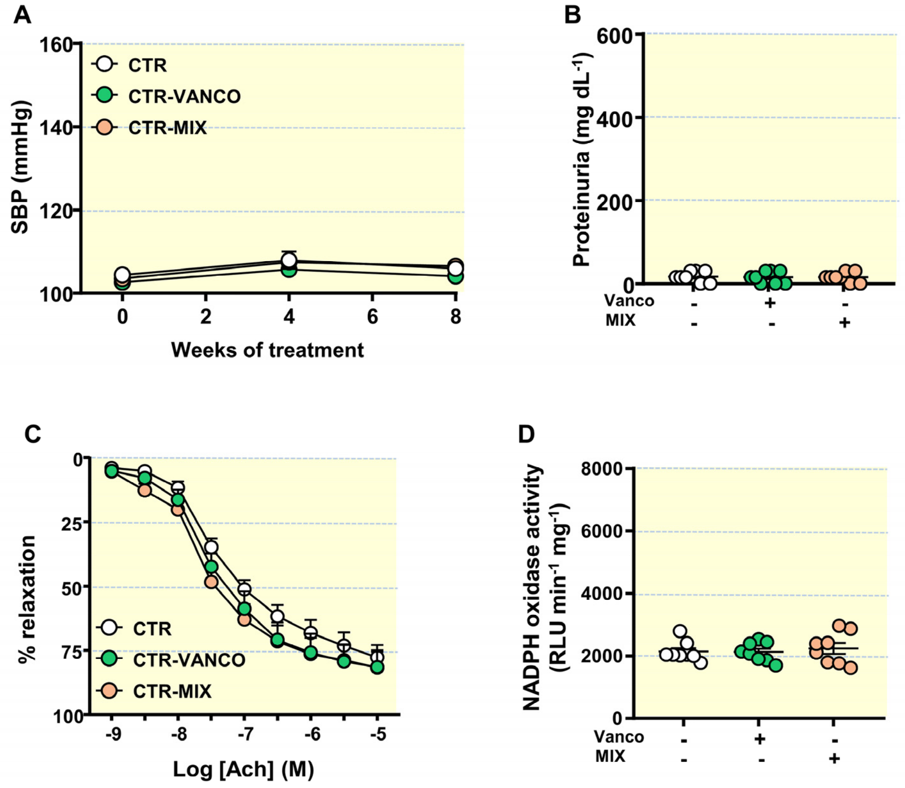

Figure S5. Effects of antibiotic treatments in blood pressure, proteinuria, and endothelial function in control (CTR) mice. (A) Systolic blood pressure (SBP) measured by tail-cuff plethysmography, (B) proteinuria, (C) vascular relaxation responses induced by acetylcholine (Ach) in endothelium-intact aortas pre-contracted by U46619 (10 nM), and (D) aortic NADPH oxidase activity measured by lucigenin-enhanced chemiluminescence, in all experimental groups. Groups: control (CTR), and CTR-groups treated with Vancomycin (VANCO) or a broad-spectrum antibiotic mixture (MIX). Values are expressed as means ± SEM

(n = 8). The concentration-response curves to Ach were analyzed by two-way ANOVA with the Tukey's multiple comparison test. The rest of the variables were tested with one-way ANOVA and Tukey *post hoc* test.

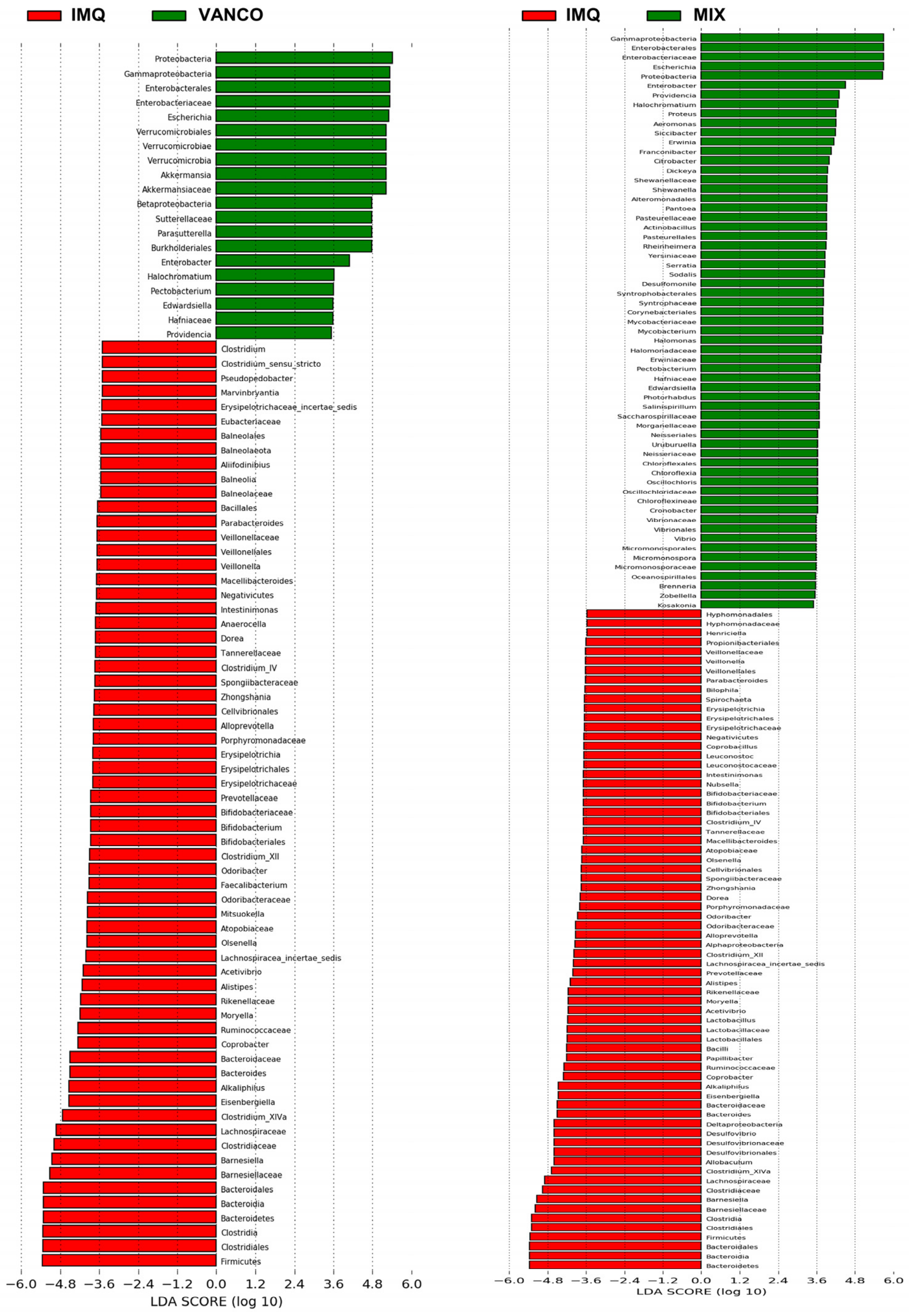

Figure S6. Effects of antibiotic treatments in taxa of gut microbiota in imiquimod (IMQ) mice. Linear discriminant analysis effect size (LEfSe) identified significantly different bacterial taxa enriched in each cohort at LDA Score > 3.5, and Kruskal-Walllis test among classes and Wilcoxon test between subclases with threshold 0.05. Groups: IMQ, and IMQ-groups treated with Vancomycin (VANCO) or a broad-spectrum antibiotic mixture (MIX).

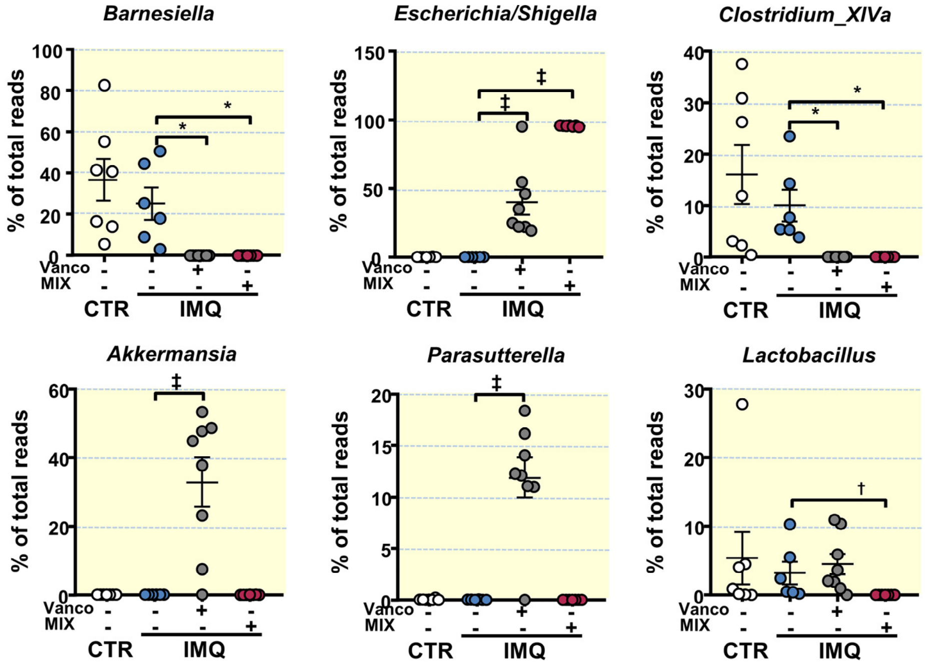

Figure S7. Effects of antibiotic treatments in the main genera proportion of gut microbiota in imiquimod (IMQ) mice. The genera content was expressed as percentage of total reads in faeces from control (CTR), IMQ and IMQ-groups treated with Vancomycin (VANCO) or a broad-spectrum antibiotic mixture (MIX). Values are expressed as means ± SEM. One-way ANOVA and Tukey *post hoc* test. *P<0.05, †P<0.01, ‡P<0.001 compared to the untreated IMQ group.

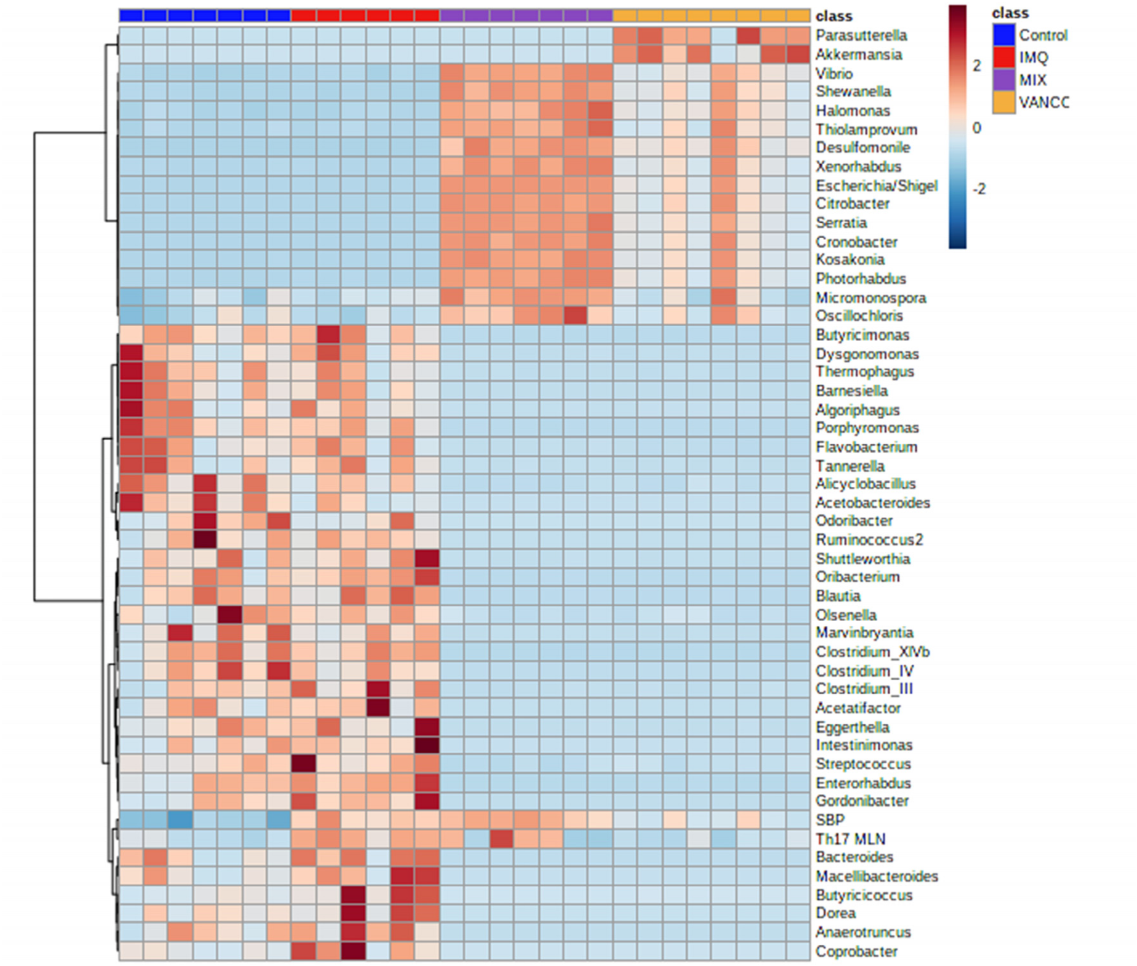

Figure S8. Effects of antibiotic treatments in the main genera proportion of gut microbiota in imiquimod (IMQ) mice associated with systolic blood pressure (SBP) and Th17 proportion in mesenteric lymph nodes (MLN). Significantly enriched genera displayed within a heat map. The top dendrogram, and top row colored blue in control (CTR), red (IMQ), violet (IMQ-group treated with Vancomycin, VANCO), and orange (IMQ-group treated with a broad-spectrum antibiotic mixture, MIX), shows that the subjects tend to cluster by SBP and Th17 in MLN (Pearson test); abundances of the genera also cluster, dendrogram on the left. Rows are genus, columns individual mouse, and abundance is shown in individual cells on a sliding color scale.

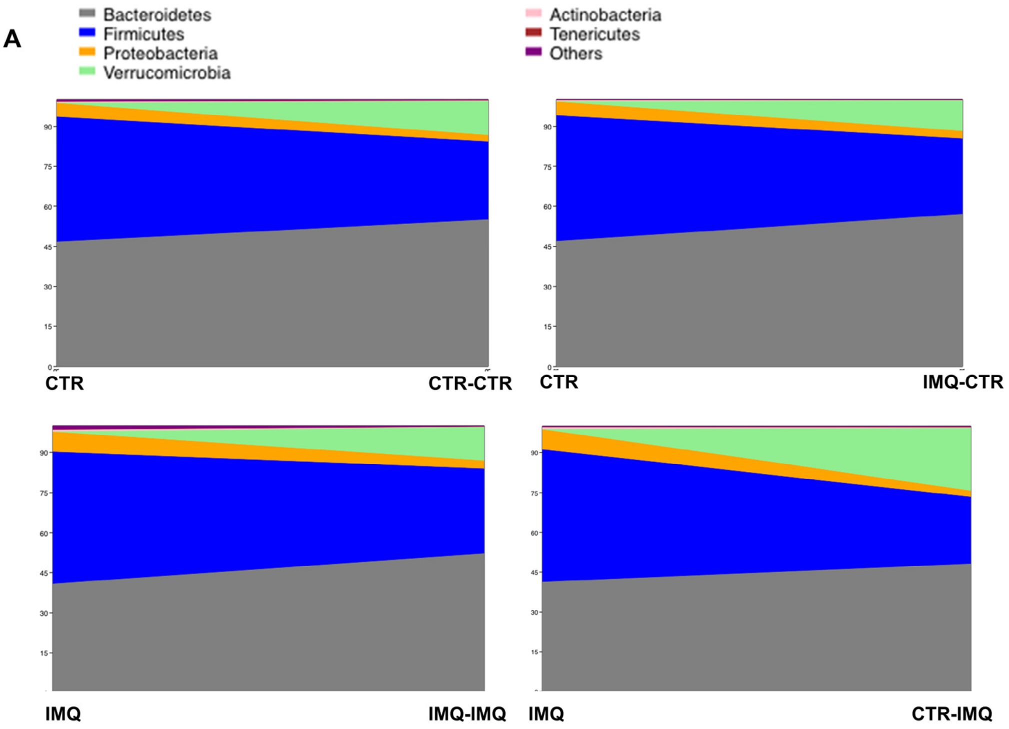

	CTR (n = 7)	CTR-CTR (n = 8)	IMQ-CTR (n = 8)	IMQ (n = 6)	IMQ-IMQ (n = 8)	CTR-IMQ (n = 8)
Actinobacteria	0.41 ± 0.07	0.15 ± 0.03†	0.09 ± 0.0‡	0.70 ± 0.11*	0.09 ± 0.03‡	0.19 ± 0.08†
Bacteroidetes	46.98 ± 11.64	55.04 ± 7.13	75.18 ± 3.43*	40.97 ± 11.04	57.06 ± 8.55	48.17 ± 5.86
Firmicutes	46.83 ± 11.61	29.21 ± 8.03	18.00 ± 2.74*	49.41 ± 10.21	28.29 ± 6.49	25.18 ± 6.54*
Verrucomicrobia	0.07 ± 0.00	12.57 ± 4.38*	3.65 ± 1.74	0.07 ± 0.00	11.25 ± 4.66*	23.48 ± 6.69†
Proteobacteria	4.97 ± 1.93	2.42 ± 0.20	2.48 ± 0.31	7.53 ± 1.13	2.81 ± 0.40‡	2.34 ± 0.23‡
Tenericutes	0.64 ± 0.21	0.18 ± 0.04*	0.15 ± 0.03*	0.08 ± 0.04	0.08 ± 0.02	0.25 ± 0.04*
Others	0.69 ± 0.19	0.34 ± 0.09	0.26 ± 0.05*	1.24 ± 0.44	0.21 ± 0.07*	0.36 ± 0.13

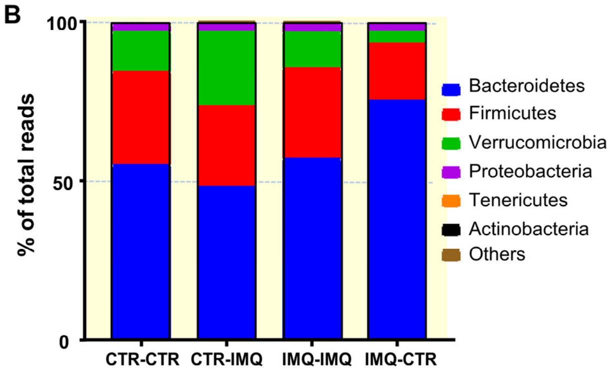

Figure S9. Phyla proportion after faecal microbiota transplantation (FMT) experiment. (A) Evolution of phyla proportion of faecal microbiota from donor control (CTR) or imiquimod (IMQ) microbiota in recipients CTR or IMQ-treated mice. (B) Final gut microbiota composition, at phyla levels, after FMT. Values are expressed as means ± SEM. unpaired t-test was used to compare two groups. *P<0.05, †P<0.01, ‡P<0.001 compared to the donor CTR group; *P<0.05, †P<0.01, ‡P<0.001 compared to the donor IMQ group. CTR-CTR, Control mice transplanted with faeces from control mice; CTR-IMQ, Control mice transplanted with faeces from IMQ-treated mice; IMQ-IMQ, IMQ-treated mice transplanted with faeces from IMQ mice; and IMQ-CTR, IMQ-treated mice transplanted with faeces from control mice.

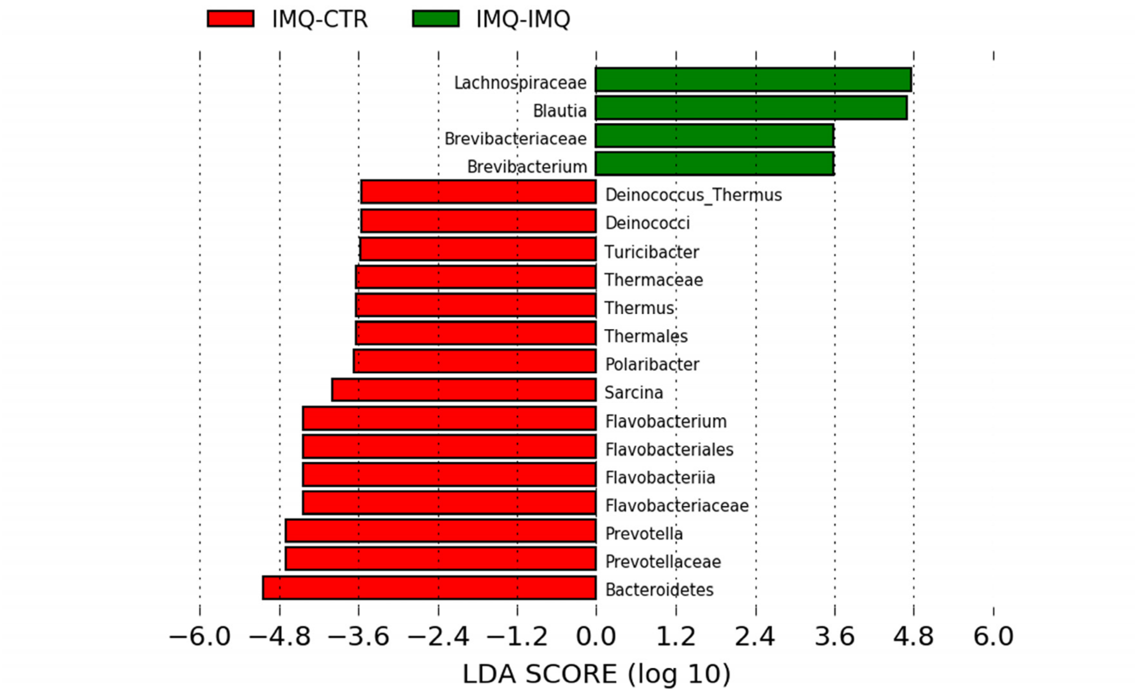

Figure S10. Effects in taxa proportion of gut microbiota after donor control (CTR) microbiota transplantation to imiquimod (IMQ)-treated mice. Linear discriminant analysis effect size (LEfSe) identified significantly different bacterial taxa enriched in each cohort at LDA Score > 3.5, and Kruskal-Walllis test among classes and Wilcoxon test between subclases with threshold 0.05 (red bars IMQ-CTR enriched, green bars IMQ-IMQ enriched). IMQ-IMQ, IMQ-treated mice transplanted with faeces from IMQ mice; and IMQ-CTR, IMQ-treated mice transplanted with faeces from control mice.

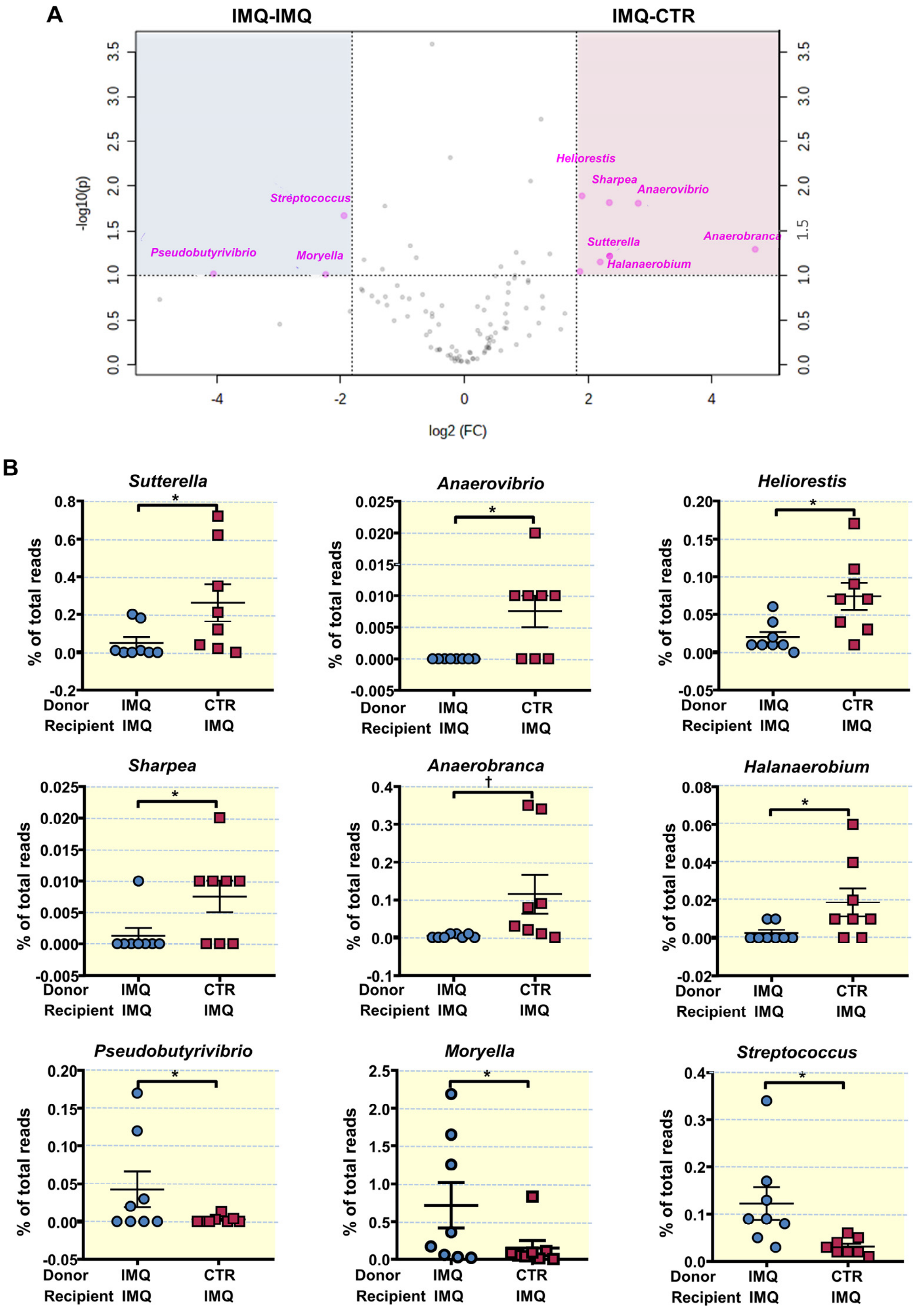

Figure 11. Effects of stool transplantation from control mice to imiquimod (IMQ)-treated mice at the genera proportion in gut microbiota**.** (A) Volcano plot displaying significantly enriched genera in IMQ-IMQ group or IMQ-CTR group. The x-axis is log2 fold change and the y-axis is transformed P-value. Genera not different between cohorts are shown in black. Features selected by volcano plot with fold change threshold 3.5 and t-test with threshold 0.05. Genera more abundant in IMQ-IMQ are shown in the upper right, blue-shaded quadrant and genera more abundant in IMQ-CTR mice in the upper left, red-shaded quadrant (B) Proportion of key bacterial genera expressed as percentage of total reads. Values are expressed as means ± SEM. Unpaired t-test. *P<0.05, †P<0.01 compared to IMQ-IMQ group. IMQ-IMQ, IMQ-treated mice

transplanted with faeces from IMQ mice; and IMQ-CTR, IMQ-treated mice transplanted with faeces from control mice.
